# *Cis*-interaction between CD52 and T cell receptor complex interferes with CD4^+^ T cell activation in acute decompensation of cirrhosis

**DOI:** 10.1016/j.ebiom.2024.105336

**Published:** 2024-09-13

**Authors:** Tong Liu, Gang Wu, Cathrin L.C. Gudd, Francesca M. Trovato, Thomas Barbera, Yan Liu, Evangelos Triantafyllou, Mark J.W. McPhail, Mark R. Thursz, Wafa Khamri

**Affiliations:** a Section of Hepatology & Gastroenterology, Division of Digestive Diseases, Department of Metabolism, Digestion & Reproduction, Imperial College London, London, United Kingdom; b Department of Life Sciences, Imperial College London, London, United Kingdom; c Department of Inflammation Biology, Institute of Liver Studies, King's College London, London, United Kingdom; d Glycosciences Laboratory, Department of Metabolism, Digestion & Reproduction, Imperial College London, London, United Kingdom

**Keywords:** Acute decompensation, Cirrhosis, Cirrhosis-associated immune dysfunction, CD4 T cells, CD52, T cell receptor

## Abstract

**Background:**

Immune dysfunction contributes to a high rate of infection in patients with acute decompensation of cirrhosis. CD52 is a glycoprotein prominently expressed in lymphocytes. Immune regulation by CD52 may be involved in adaptive immune dysfunction in cirrhosis. This study aimed to investigate the function of CD52 on CD4^+^ T cells on the blood of patients with acute decompensation of cirrhosis.

**Methods:**

The expression of CD52 in the peripheral blood lymphocytes of 49 patients with cirrhosis was investigated using flow cytometry and transcriptomics. Potential *cis*-membrane ligands of CD52 were discovered via proximity labelling followed by proteomics. The function of CD52 on antigen-specific activation of CD4^+^ T cells was examined using flow cytometry in CD52 CRISPR-Cas9 knockout primary T cells.

**Findings:**

CD52 expression was elevated in CD4^+^ T cells in acute decompensation of cirrhosis, and this elevation was correlated with increased disease severity and mortality. Components of the T cell receptor complex including TCRβ, CD3γ and CD3ε were identified and validated as *cis*-membrane ligands of CD52. Knockout of CD52 promoted antigen-specific activation, proliferation, and pro-inflammatory cytokine secretion.

**Interpretation:**

Membrane bound CD52 demonstrated *cis*-interaction with the T cell receptor and served as a dynamic regulator of antigen-specific activation of CD4^+^ T cells. The upregulation of CD52 in the periphery of acute decompensation of cirrhosis hinders the recognition of the T cell receptor by MHC, contributing to impaired T cell function. The development of an alternative anti-CD52 antibody is required to restore T cell function and prevent infections in cirrhosis.

**Funding:**

This study was supported by the 10.13039/501100013342NIHR Imperial Biomedical Research Centre, Institute for Translational Medicine and Therapeutics (P74713), 10.13039/100010269Wellcome Trust (218304/Z/19/Z), and 10.13039/501100000265Medical Research Council (MR/X009904/1 and MR/R014019/1).

**Research in context**

**Evidence before this study**

T cell dysfunction contributes to disturbed immune response in acute decompensation of cirrhosis. CD52, a suppressive marker with no clear evidence on the mechanism of suppression was found to be upregulated in an expanded CD4^+^HLA-G^+^ suppressor T cell population found in these patients.

**Added value of this study**

CD52 expression is elevated in CD4^+^ T cells in the periphery of acute decompensation of cirrhosis. Proteins TCRβ, CD3ε and CD3γ have *cis*-interaction with CD52 on the membrane of CD4^+^ T cells. CD52 interferes with the T cell receptor and prevents antigen-specific activation of CD4^+^ T cells. Elevated CD52 contributes to adaptive immune dysfunction in acute decompensation of cirrhosis.

**Implications of all the available evidence**

Upregulated CD52 in CD4^+^ T cells suppresses antigen-specific T cell responses in acute decompensation of cirrhosis and might contribute to the susceptibility to infection observed in these patients. Targeting CD52 could reverse this suppression, but the currently available monoclonal antibody alemtuzumab is not suitable for this purpose. The development of a novel antibody targeting CD52 without T cell depletion could be a potential direction for immune therapy to prevent infections in cirrhosis.

## Introduction

Dynamic control of T cell activation is a critical component of antigen-specific immune responses against pathogens. The balance between activating and inhibitory signals dictates the level of T cell activation in response to antigen exposures. Central to the antigen-specific T cell response is the activation of the T cell receptor (TCR), the regulation of which can be modulated by interfering with the TCR signalling cascade with immune checkpoint receptors such as PD-1, CTLA-4, and TIGIT.^1^, ^2^, ^3^ These receptors rely on ligands expressed in antigen-presenting cells (APCs) or other regulatory cells. Regulation of activation without ligands from neighbouring cells is also possible via *cis*-interactions between the receptors and their ligands.^4^ The *cis*-interactions between CD2 and CD48/CD58 have recently been reported to play a vital role in the initiation of TCR signalling in T cells,^5^ while the regulation of the inhibitory receptor CD22 by its *cis*-ligands in B cells is another well-known example.^5^, ^6^, ^7^

Progressive dysfunction of immune responses is commonly associated with the onset of acute decompensation of cirrhosis (AD), known as cirrhosis-associated immune dysfunction: concomitant or alternation between systemic inflammation and defective immune response to pathogens.^8^^,^^9^ The latter is responsible for the heightened susceptibility to infections in patients with decompensated cirrhosis,^10^, ^11^, ^12^ with adaptive immune dysfunction in AD attracting growing research interest.^13^^,^^14^ Immune modulation by regulatory T cells (Tregs) plays a crucial role in balancing the pathogen-specific immune responses and autoimmunity.^15^ Dysregulation in multiple subsets of Tregs, including both conventional thymus-derived Tregs and less conventional peripherally-induced Tregs, has been reported in the peripheral blood of patients with cirrhosis and AD.^16^, ^17^, ^18^, ^19^, ^20^, ^21^ Our previous study identified an expanded CD4^+^HLA-G^+^IL-35^+^ suppressive T cell population in AD which was transcriptomically characterised by the high expression of CD52.^21^

CD52 is a small glycoprotein abundantly expressed on the surface of lymphocytes and is found in 95% of the peripheral T and B cells.^22^, ^23^, ^24^ Therefore, CD52 has been widely used as a target for monoclonal antibodies in the treatment of lymphoproliferative diseases.^25^^,^^26^ Compared to the long history of application of the CD52 monoclonal antibody alemtuzumab, the function of CD52 on T cells was not discovered until recently. Being glycosylphosphatidylinositol-anchored and lacking a transmembrane structure, CD52 does not have a direct downstream signalling pathway, but can be cleaved by phospholipase C and released in a soluble form.^22^^,^^24^^,^^27^ Soluble CD52 can then serve as the counter-receptor for inhibitory sialic acid-binding immunoglobulin-like lectin 10 (Siglec-10) which inhibits T cell proliferation and activation through its immunoreceptor tyrosine-based inhibitory motif.^28^^,^^29^ Studies have shown that cross-linking of CD52 on T cells results in enhanced T cell proliferation.^30^^,^^31^ Evidence suggests that the signal transduction of this effect is TCR- and CD45-dependent, however, the exact mechanism remains unknown.^32^ This study aimed to investigate the function of CD52 in the peripheral T cells of patients with AD.

## Methods

### Patient characteristics

This study recruited 49 patients with cirrhosis between May 2018 and January 2021, including AD (n = 28, defined as patients presenting to the hospital with acute decompensation with or without organ failure) and chronic decompensated cirrhosis (CD, n = 21, encompassing both unstable decompensated cirrhosis necessitating readmission and stable decompensated cirrhosis requiring elective procedures), in line with the PREDICT study definitions.^33^ The patient clinical parameters are presented in Table 1. Detailed patient criteria are described in the Supplemental Materials. Healthy volunteers (n = 17) were recruited as healthy controls (HC). The sample size was determined based on previously reported CD52 MFI in CD4^+^ T cells,^34^^,^^35^ assuming a 20% alteration, with a power of 90% and alpha of 0.05, requiring a minimum of 11 patients per group. Power calculation undertaken in Medcal version 22 software (Medcalc Software, Ostend, Belgium).

**Table 1:** Demographics and clinical parameters of patients with AD, CD and HCs.

Parameter	AD (n = 28)	CD (n = 21)	HC (n = 17)
Age―years	51.50 (44.00–57.00)	54.00 (47.00–62.00)	32.00 (30.00–36.00)
Sex―n (%)			
Female	8/28 (28.57%)	5/21 (23.81%)	6/17 (35.29%)
Male	20/28 (71.43%)	16/21 (76.19%)	11/17 (64.71%)
Aetiology―n (%)			
Alcohol related liver disease	20/28 (71.43%)	12/21 (57.14%)	N/A
Metabolic associated fatty liver disease	5/28 (17.86%)	2/21 (9.52%)	N/A
Primary biliary cholangitis	1/28 (3.57%)	2/21 (9.52%)	N/A
Primary sclerosing cholangitis	–	3/21 (14.29%)	N/A
Cryptogenic	2/28 (7.14%)	2/21 (9.52%)	N/A
Leukocytes— × 10^9^/L	8.47 (6.64–16.46)	4.57 (2.67–6.33)	N/A
Neutrophils— × 10^9^/L	6.70 (4.48–13.35)	2.53 (1.97–3.98)	N/A
Monocytes— × 10^9^/L	0.80 (0.50–1.13)	0.34 (0.23–0.49)	N/A
Lymphocytes— × 10^9^/L	1.08 (0.79–1.44)	1.00 (0.75–1.43)	N/A
Platelet — × 10^9^/L	104.00 (48.25–142.00)	120.00 (68.00–160.00)	N/A
Bilirubin—μmol/L	144.50 (77.50–399.75)	44.00 (25.00–131.00)	N/A
Creatinine—μmol/L	80.50 (62.50–131.75)	68.00 (54.00–88.00)	N/A
CRP—mg/L	28.65 (9.50–58.28)	8.20 (3.20–17.10)	N/A
INR	1.87 (1.57–2.27)	1.43 (1.21–1.73)	N/A
AD with ACLF	13/28 (46.43%)	N/A	N/A
CLIF AD score (in AD) CLIF ACLF (in ACLF)	52.00 (46.50–60.00) 62.00 (52.00–65.00)	N/A	N/A
Child-Pugh score	10.50 (9.00–11.25)	8.00 (7.00–10.00)	N/A
MELD-Na score	45.00 (34.25–56.50)	18.00 (15.00–24.00)	N/A
SOFA score	7.00 (4.75–10.25)	4.00 (3.00–4.00)	N/A

### Flow cytometry phenotyping

Surface marker staining of peripheral blood mononuclear cells (PBMC) was conducted using fluorochrome-labelled monoclonal antibodies (Supplemental Table S1), as detailed in the Supplemental Materials.

### Cell sorting and transcriptomics profiling

Viable CD3^+^CD4^+^CD8^−^ T cells from patients with AD (n = 4) were randomly selected from available samples and sorted using a FACS Aria II flow cytometer (Becton Dickinson, Oxford, UK) into Tregs and effector T cells (gating strategy described in the Supplemental Materials). The NanoString nCounter GX Human Immunology V2 assay (NanoString Technologies, WA, USA) was carried out as described in the Supplemental Materials.

### CD4^+^ T cell isolation and gene expression assay

CD4^+^ T cells were isolated from PBMCs via negative selection, employing magnetic-activated cell sorting with a human CD4^+^ T cell isolation kit (Miltenyi Biotec, Surrey, UK), according to the manufacturer's instructions. RNA extraction and reverse transcription are described in the Supplemental Materials. The expression of *CD52* gene was assessed by TaqMan gene expression assay with a *CD52* probe (Hs00174349_m1) (Thermo Fisher Scientific, MA, USA), and human *GAPDH* (Hs02786624_g1) was used as the endogenous control. Quantitative amplification was performed according to the manufacturer's instructions using a Step One Plus Real-Time PCR System (Thermo Fisher Scientific). Gene expression levels were normalised to *GAPDH* and expressed as a fold-change (ratio of 2^−ΔΔCT^).

### Proximity labelling and quantitative proteomics

For CD52 proximity labelling, 2 × 10^6^ magnetic beads-isolated AD CD4^+^ T cells (n = 4) were stained with horseradish peroxidase (HRP)-conjugated mouse anti-human CD52 monoclonal antibody or mouse IgG1 isotype (R&D Systems, Abingdon, UK). The stained cells were incubated with 95 μM tyramide-SS-biotin (Iris Biotech, Germany) and 0.01% (v/v) hydrogen peroxide (Sigma–Aldrich, Dorset, UK) on ice for 2 min. The reaction was terminated using 3 mL of 100 U/mL catalase (Sigma–Aldrich). Cells were then lysed, and the biotinylated proteins were enriched on streptavidin Dynabeads (Thermo Fisher Scientific), which were then eluted by dithiothreitol reduction. The eluted proteins were processed into peptides using the SP3 paramagnetic beads method for quantitative proteomic analysis.^36^ Detailed protein purification and data analysis methods are described in the Supplementary Materials.

### Protein avidity measurement

The recombinant human CD52 (rCD52) protein (R&D Systems) was labelled with Alexa Fluor 488 dye using a Thermo Fisher Alexa Fluor labelling kit (Thermo Fisher Scientific). Recombinant human TCRβ1, CD3ε, CD3γ, CD43, CD44, CD48 (Cusabio Biotech), CD4 (ABclonal, Port Talbot, UK), integrin β2 (Assay Genie, Dublin, Ireland) proteins, recombinant human CD52 monoclonal antibody, Siglec-10, and IgG1 proteins (R&D Systems) were labelled with 4-((4-(dimethylamino)phenyl)azo)benzoic acid (DABCYL) using DABCYL-succinimidyl ester. Detailed labelling methods and recombinant protein sequences are described in Supplemental Materials (Supplemental Table S2). Alexa Fluor 488-conjugated rCD52 protein was reconstituted in 0.1M pH9.6 sodium carbonate-bicarbonate buffer at 10 μg/ml and immobilised on polystyrene high binding surface plates (Corning, NY, USA). Immobilised rCD52 was probed with DABCYL-conjugated protein at 0.125–1 μM in Dulbecco's phosphate-buffered saline (Thermo Fisher Scientific). The plate with rCD52 was pre-treated with 16 mU/mL *Vibrio cholerae* sialidase, 1 mM EDTA (Sigma–Aldrich), or buffer control. Fluorescence was measured at 485 nm excitation and 520 nm emission wavelengths using a FLUOstar OPTIMA spectrophotometer (BMG Labtech, Germany).

### Immunoprecipitation

Pooled magnetic bead-isolated CD4^+^ T cells from patients with AD (n = 3) were lysed with NP40 buffer (Thermo Fisher Scientific). Immunoprecipitation detected by flow cytometry (IP-FCM) was adapted from Davis et al.^37^ CD52 monoclonal antibodies (alemtuzumab biosimilar) (R&D Systems), CD3 monoclonal antibodies (Thermo Fisher Scientific), or IgG1 isotype were captured on protein G Dynabeads (Thermo Fisher Scientific) and incubated with cell lysate, in a non-denatured environment. Bead-protein complexes were detected by flow cytometry using fluorescein isothiocyanate (FITC)-conjugated CD3ε, CD4, TCRβ (Thermo Fisher Scientific) or CD52 (Miltenyi Biotec) monoclonal antibodies.

### CD4^+^ T cell Förster resonance energy transfer flow cytometry and microscopy

Magnetic bead-isolated CD4^+^ T cells were nuclei stained with SYTO™ 59 (Thermo Fisher Scientific), surface stained with FITC-conjugated CD3ε, CD4, TCRβ or CD5 (BioLegend, CA, USA) monoclonal antibodies and probed with DABCYL-conjugated recombinant human CD52 monoclonal antibody or recombinant human IgG1 protein, in Hanks' balanced salt solution (Thermo Fisher Scientific), with or without 1 mM EDTA. Then, cells were fixed with 4% (w/v) paraformaldehyde and fluorescence was detected using flow cytometry or fluorescence confocal microscopy, as detailed in the Supplemental Materials.

### CD52 and ligands AlphaFold model

The amino acid sequences of human CD52, CD3ε, CD3γ and TCRβ1 were submitted to AlphaFold Multimer to produce five models of the complex.^38^ The top ranked model was visualised using UCSF ChimeraX, version 1.6.1.^39^

### T cell proliferation assay

Magnetic bead-isolated CD4^+^ T cells from HCs (n = 5) and patients with AD (n = 5) were randomly selected from available samples and underwent CRISPR knockout of CD52 using a ribonucleoprotein electroporation method^40^ and were co-cultured with allogeneic monocyte-derived dendritic cells (MoDCs, at 10:1 T cell to MoDC ratio, at 37 °C in 5% CO2. Detailed protocols for CD52 knockout (KO) and MoDC differentiation are described in the Supplementary Materials. Prior to co-culture, CD4^+^ T cells were stained with 10 μM Cell Proliferation Dye (CPD) eFluor 670 (Thermo Fisher Scientific) according to the manufacturer's protocol. Cells were cultured in serum-free TexMACS medium for 6 days after stimulation with 5ug/ml CEFT MHC-II peptide pool (Supplemental Table S4) (ProImmune Ltd, Oxford, UK), 10 ug/ml CD52 monoclonal antibodies, 10 ug/ml recombinant human CD52 (R&D Systems), or 5 ug/ml CD3 antibodies. Activation of gated CD4^+^ T cells was measured using flow cytometry on days 3 and 6. Proliferation of gated CD4^+^ T cells was measured by dilution of the CPD dye using flow cytometry, as detailed in the Supplemental Materials (Supplemental Table S5). Supernatants were collected to assess interferon-γ (IFN-γ) and interleukin-2 (IL-2) cytokine secretion using S-PLEX Human IL-2 and U-PLEX Human IFN-γ electrochemiluminescence assays (Meso Scale Discovery System, MD, USA).

### Statistics

Statistical analyses were performed using GraphPad Prism version 8.02 software (GraphPad Software, San Diego, USA). D'Agostino-Pearson normality test was performed to determine normal distribution. Correlations were tested using Spearman's rank correlation coefficient. Categorical data was compared using Fisher's exact test. Nonparametric data between two groups were compared with Mann–Whitney U test if unpaired, or Wilcoxon Signed-Rank test if paired. Nonparametric data between more than two groups were compared with Kruskal–Wallis test if unpaired, Friedman test if paired, followed by multiple comparisons with two-stage step-up method of Benjamini, Krieger and Yekutieli.

### Ethics

This study was approved by the regional research ethic committees North West Haydock REC 19/NW/0750 and Wales REC3 17/WA/0161. Study was performed in accordance with the Declaration of Helsinki, informed consent was obtained from all subjects.

### Role of funders

Funders were not involved in study design, data collection, data analyses, interpretation, or writing of report.

## Results

### Patient characteristics

The age and sex distributions were statistically balanced between the AD and CD groups; however, both groups were significantly older than the HC group (p < 0.01, p < 0.001, respectively, Fisher's exact test) (Table 1). There were no significant differences in sex proportions. In both groups, the most common aetiology of liver disease was alcohol-related liver disease (71% in AD and 57% in CD), followed by metabolic-associated fatty liver disease (18% in AD and 10% in CD). Leukocyte count, total bilirubin, C reaction protein (CRP) and international normalised ratio (INR) levels were significantly higher in the AD group than in the CD group (p < 0.001, p < 0.05, p < 0.01, and p < 0.01, respectively, Mann–Whitney test). Child-Pugh score, MELD-Na score, and SOFA score were also significantly elevated in patients with AD compared to those with CD (p < 0.05, p < 0.001, and p < 0.001, respectively, Mann–Whitney test). Among all patients with AD, 46% had extra-hepatic organ failure and were defined as acute-on-chronic liver failure (ACLF).

### Upregulation of CD52 in CD4^+^ T cells in acute decompensation of cirrhosis

**Fig. 1:**
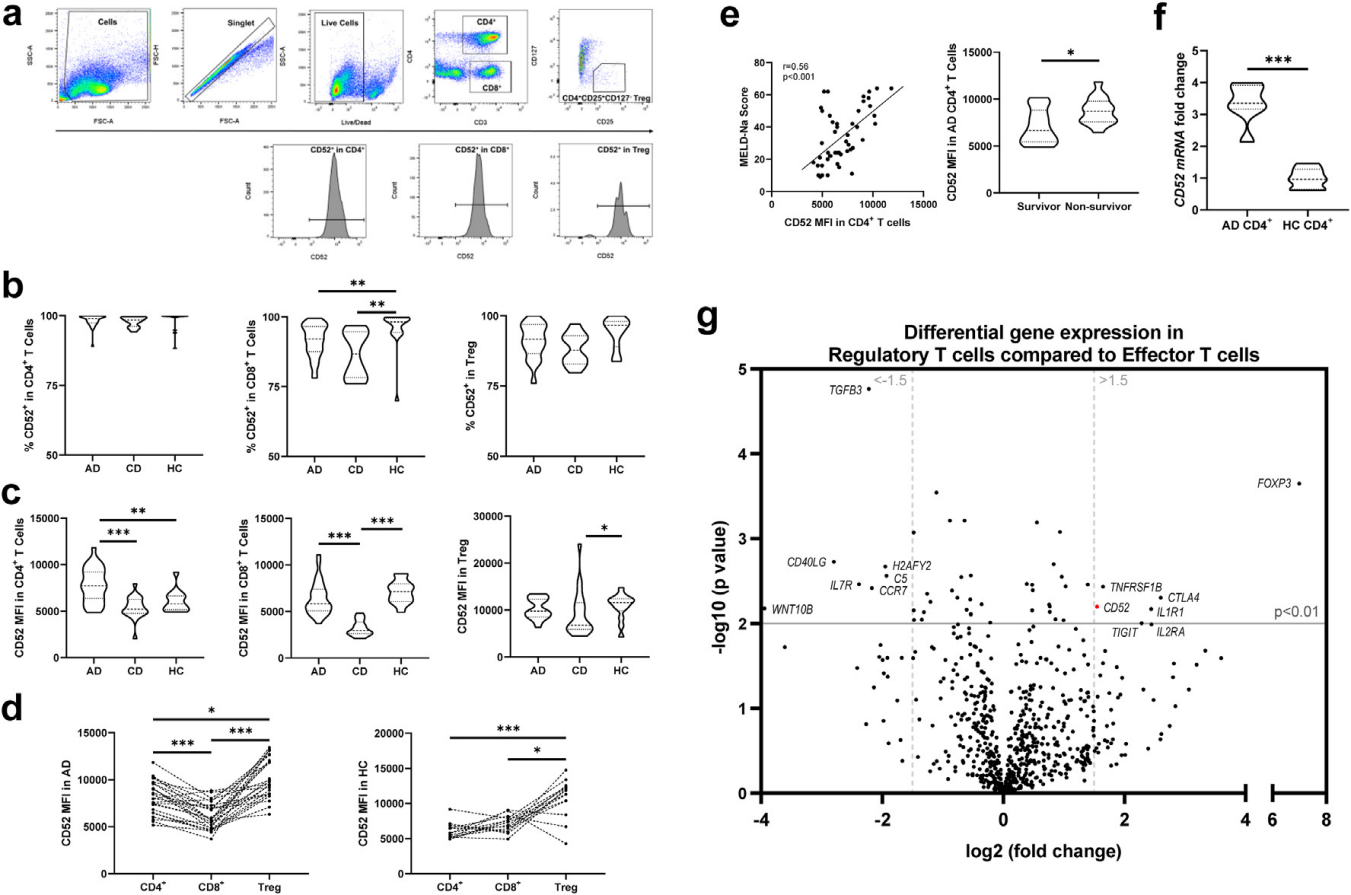
**CD52 expression in T cell populations in patients with AD, CD and HC**. PBMCs from patients (AD, n = 28; CD, n = 21) and HC (n = 17) were assessed for surface levels of CD52 expression using flow cytometry. **(a)** Gating strategy and representative flow cytometry histograms used to determine levels of CD52, all gating based on fluorescence-minus-one (FMO) controls. **(b)** Percentage of CD52 expressing cells in CD4^+^ T cells (left panel), CD8^+^ T cells (middle panel) or CD4^+^CD25^+^CD127^-^ Tregs (right panel) in patients with AD or CD, and HC. **(c)** MFI of CD52 in CD52^+^CD4^+^ T cells (left panel), CD52^+^CD8^+^ T cells (middle panel) or CD52^+^ Tregs (right panel) in patients with AD or CD, and HC. **(d)** Comparisons of CD52 MFI in CD52^+^ cells among CD4^+^ T cells, CD8^+^ T cells and Tregs, in patients with AD (left panel) or HC (right panel). **(e)** Correlation between CD52 MFI in CD4^+^ T cells and MELD-Na score in patient samples (left panel). Distribution of CD52 MFI in CD4^+^ T cells in non-surviving (deceased or transplanted, n = 14) and surviving patients (n = 14) with AD within 90 days following admission (right panel). **(f)** CD4^+^ T cells were isolated from PBMCs of patients with AD (n = 7) and HC (n = 7). Relative expression of *CD5*2 mRNA was measured by real-time PCR. Data expressed as fold-change to HC CD4^+^ average *CD52* expression. **(g)** Quantitative transcriptomics analysis of immune-related gene in Tregs and effector T cells from patients with AD (n = 4) using NanoString Technologies. Volcano plots comparing Treg to effector T cells. Gene names are listed for differentially expressed genes. Non-parametric statistical analysis was used (Mann–Whitney test for two group comparison, Kruskal–Wallis test for unpaired data, or Friedman test for paired data, followed by multiple comparisons with two-stage step-up method of Benjamini, Krieger and Yekutieli for multiple comparisons between more than two groups). Data are presented as median values with interquartile range (IQR). Correlation coefficient (r) and p value were tested using Spearman's correlation test. ∗p < 0.05 ∗∗p < 0.01 ∗∗∗p < 0.001.

PBMCs from 28 patients with AD, 21 patients with CD, and 17 HCs underwent phenotypic evaluation for CD52 expression in CD4^+^ T cell, CD8^+^ T cell and CD4^+^CD25^+^CD127^-^ Treg populations, flow cytometry gating strategies are described in Fig. 1a. Data revealed the expression of CD52 in all T lymphocyte sub-populations regardless of the disease group (Fig. 1b). CD52 expression in CD4^+^ T cells was ubiquitous, with no significant difference in the percentage expression observed among the AD, CD, and HC groups. In CD8^+^ T cells, AD and CD showed slightly lower percentage expression compared to HC [92.10% (87.50–96.58%) vs. 86.70% (78.25–94.70%) vs. 98.20% (94.40–99.50%)]. No difference in the percentage expression of CD52 in Tregs was found among the AD, CD, and HC groups. There was no difference in CD52 expression between patients with AD with and without ACLF (Supplemental Figure S1a).

Despite the ubiquitous high percentage expression, the medium fluorescence intensity (MFI) of CD52 differed between the disease groups (Fig. 1c). In CD4^+^ T cells, CD52 MFI in AD was significantly elevated compared to that in CD and HC [7704 (6352–9209) vs. 5224 (4786–6267), p < 0.001 vs. 5785 (5157–6629), p < 0.01, Kruskal–Wallis test]. This elevation was independent of age (Supplemental Figure S1g-i). In the CD8^+^ T cell compartment, CD52 MFI in AD was found to be elevated compared to that in CD but not to HC [5832 (5087–7376) vs. 2946 (2626–3881), p < 0.001 vs. 7118 (6059–7965)]. CD52 MFI was not altered in AD Tregs [9799 (8512–12348), 6763 (5919–11586), 11,577 (9874–12381), in AD, CD, and HC, respectively]. Looking among T cell compartments, Tregs showed an elevation of CD52 MFI compared to CD4^+^ or CD8^+^ T cells in HC (p < 0.001, p < 0.01, respectively, Friedman test) and in AD (p < 0.05, p < 0.001, Friedman test) (Fig. 1d). In the AD and CD cohorts, the CD52 MFI in CD4^+^ T cells was positively correlated with the MELD-Na score (Fig. 1e). Sub-analysis of patients with AD based on 90-day mortality revealed that survivors had significantly lower CD52 MFI in CD4^+^ T cells than patients who died or received liver transplant within 90 days post-admission. In addition, patients who developed culture-positive infections had high CD52 MFI in CD4^+^ T cells than patients who were culture-negative, the CD52 MFI in CD4^+^ T cells was also correlated with serum C-reaction protein (CRP) (Supplemental Figure S1e & f). Siglec-10, a known ligand of CD52, showed higher expression in CD4^+^ T cells than in CD8^+^ T cells and Tregs, while CD52 expression in CD4^+^ T cells was elevated in AD compared to that in CD or HC (Supplemental Figure S1b). While HCs were significantly younger than patients with AD and CD, CD52 MFI in CD4^+^ T cells did not vary with age (Supplemental Figure S1g & h).

We also measured *CD5*2 mRNA expression using RT-qPCR in CD4^+^ T cells isolated from a different cohort of patients with AD and HCs (n = 7 in each group) (Fig. 1f). In patients with AD, *CD52* expression was significantly upregulated when compared to that in HCs [0.96-fold change (0.65–1.28) vs. 3.35 (3.17–3.91), p < 0.001]. Subsequently, gene expression profiling was conducted for Tregs and effector CD4^+^ T cells (Treg-depleted CD4^+^ T cells) in patients with AD (Fig. 1g and Supplemental Table S6). As anticipated, Tregs exhibited a unique gene expression pattern distinct from that of effector CD4^+^ T cells, characterised by the upregulation of signature Treg genes such as *FOXP3* and *IL2RA* (CD25), and downregulation of *IL7R* (CD127) and *TGFB*. In addition, *CD52* was also upregulated in Tregs (2.92-fold change, p < 0.01). The transcription level results were corroborating our observations at the protein expression level: CD52 expression was significantly higher in Tregs than in effector CD4^+^ T cells; CD52 expression in CD4^+^ T cells was elevated in AD, reaching the level seen in Tregs. This elevation was not observed in CD8^+^ T cells.

### Identification of potential *cis*-ligands of CD52

**Fig. 2:**
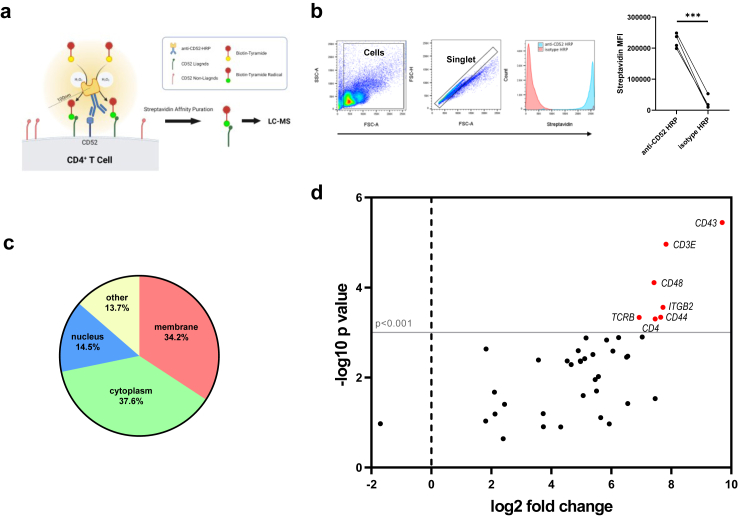
**Proximity labelling discovers potential *cis*-membrane ligands of CD52**. **(a)** CD4^+^ T cells were magnetically isolated from PBMCs of patients with AD (n = 4) and underwent proximity labelling, purification of biotinylated proteins and LC-MS quantitative proteomics. **(b)** Biotinylation of cells using anti-CD52-HRP was confirmed with flow cytometry (right panel). Representative flow cytometry histograms used to determine levels of biotin (left panels). **(c)** GOCC analysis of 117 proteins identified by quantitative proteomics. **(d)** Volcano plots showing 40 membrane proteins identified by quantitative proteomics. Gene names are listed for differentially expressed proteins. Paired t test was used for two group comparison. Data are presented as median values with IQR. ∗p < 0.05.

To label the potential ligands of CD52, CD4^+^ T cells were isolated from four patients with AD and a proximity labelling technique was employed.^34^ The membrane CD52 molecules were tagged with an HRP-conjugated anti-CD52 antibody. HRP-conjugated isotype antibody was used as the negative control. HRP catalyses the radicalisation of biotin-conjugated tyramide and produces short-lived biotin-tyramide radicals, which covalently label proteins within a 20–100 nm radius of CD52 (Fig. 2a). Biotin labelling was confirmed via flow cytometry using APC-conjugated streptavidin: cells probed with HRP-conjugated anti-CD52 antibody demonstrated higher streptavidin affinity than those treated with the HRP-conjugated isotype control antibody (Fig. 2b). Biotinylated proteins were isolated and subsequently identified by LC-MS-based quantitative proteomics. Quantitative proteomics led to the identification of 117 proteins (Supplemental Figure S2). Gene ontology-cellular component (GOCC) analysis revealed that 40 of these proteins were membrane proteins (Fig. 2c). Seven proteins: CD43, CD3ε, CD48, integrin β2, CD44, TCRβ and CD4, fell within the significance cut-off range (p < 0.001) (Table 2).

**Table 2:** Membrane proteins identified by proximity labelling.

Gene name	Protein name	Log2 fold change	p value
CD43	CD43/leukosialin	9.697	0.00000359
CD3E	CD3 ε chain	7.827	0.0000109
ITGB2	integrin subunit β2	7.725	0.000277
CD44	CD44	7.646	0.000459
CD4	CD4	7.464	0.000501
CD48	CD48	7.423	0.0000779
TCRB	T cell receptor β chain	6.928	0.000461

### CD3ε, CD4 and TCRβ interact with CD52

**Fig. 3:**
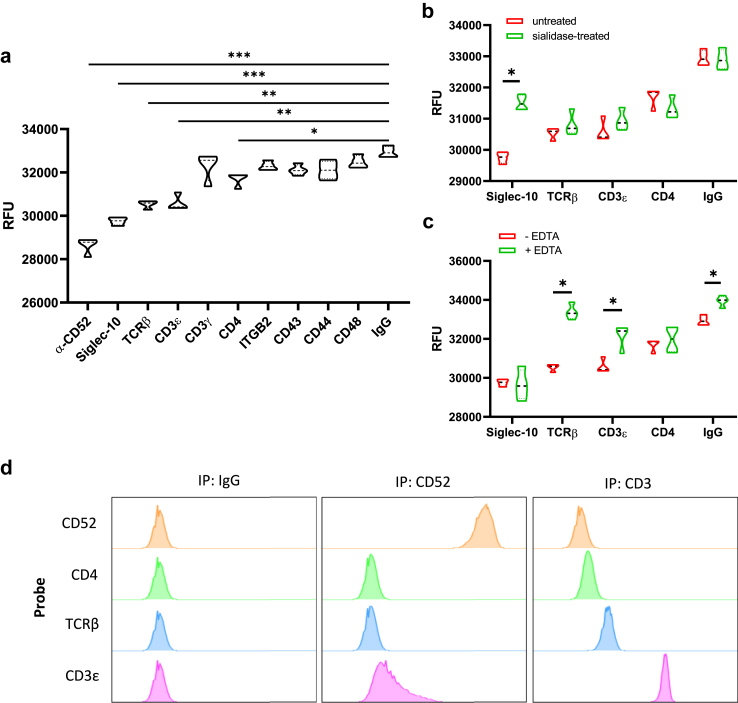
**Avidity between CD52 and potential ligands**. **(a)** Fluorescence from immobilised Alexa Fluor 488-conjugated recombinant CD52 proteins was measured after probing with DABCYL-conjugated recombinant Siglec-10, TCRβ, CD3ε, CD3γ, CD4, ITGB2, CD43, CD44 or CD48 proteins, as well as anti-CD52 antibody and human IgG. **(b)** Fluorescence from immobilised Alexa Fluor 488-conjugated, sialidase treated recombinant CD52 proteins was measured after probing with DABCYL-conjugated recombinant Siglec-10, TCRβ, CD3ε and CD4 proteins, as well as human IgG. **(c)** Fluorescence from immobilised Alexa Fluor 488-conjugated recombinant CD52 proteins was measured after probing with DABCYL-conjugated recombinant Siglec-10, TCRβ, CD3ε and CD4 proteins, as well as human IgG, with or without the presence of 1 mM EDTA. **(d)** Immunoprecipitation detected by flow cytometry on pooled CD4^+^ T cell lysate. Immunoprecipitation (IP) by CD52 or CD3 antibodies or IgG isotype, probed with FITC-conjugated CD52, CD4, TCRβ or CD3ε antibodies. RFU, relative fluorescence units. Non-parametric statistical analysis was used (Mann–Whitney test for two-group comparison and Kruskal–Wallis test followed by multiple comparisons with a two-stage step-up method of Benjamini, Krieger and Yekutieli for multiple comparisons between more than two groups). Data are presented as median values with IQR. ∗p < 0.05 ∗∗p < 0.01 ∗∗∗p < 0.001.

To further confirm the avidity between potential CD52 ligands and the CD52 molecule, the extracellular domain of CD52, conjugated with Alexa Fluor 488 fluorescent dye, was immobilised on polystyrene plates and probed with DABCYL quencher-conjugated candidate proteins. The fluorescence intensity was measured using a spectrophotometer. The Förster resonance energy transfer (FRET) pair of Alexa Fluor 488 and DABCYL has a Förster radius of 4.9 nm.^41^ Anti-CD52 antibody and Siglec-10, a known ligand of CD52, were used as the positive controls. Only TCRβ, CD3ε, and CD4, which are all components of the TCR complex, exhibited a significant FRET effect, leading to quenching of CD52 fluorescence (Fig. 3a and Supplemental Figure S3a). The Siglec-10-CD52 interaction is mediated by the recognition of sialic acid-terminating glycans on CD52 by the lectin domain of Siglec-10; this glycan-mediated interaction is characterised by low-affinity and high avidity and can be disrupted by sialidase digestion of sialic acids. To investigate whether a similar interaction exists between TCRβ, CD3ε, CD4 and CD52, plate-bound CD52 was pre-treated with sialidase before probing. The quenching by Siglec-10 was reversed by sialidase treatment, as expected, while fluorescence intensity after TCRβ, CD3ε, and CD4 probing did not increase, suggesting that their binding avidities were not affected by sialic acid removal (Fig. 3b). The quenching of CD52 fluorescence by TCRβ, CD3ε and CD4 was reversed by EDTA (Fig. 3c). IP-FCM was used to isolate the CD52-ligand complex from the CD4^+^ T cells. However, CD4 and TCRβ were not detected, and CD3ε exhibited only a very weak signal, suggesting that CD52 and its ligands had weak or transient interactions and could not be pulled down by immunoprecipitation (Fig. 3d).

### *Cis*-interactions between CD52 and TCR on CD4^+^ T cells

**Fig. 4:**
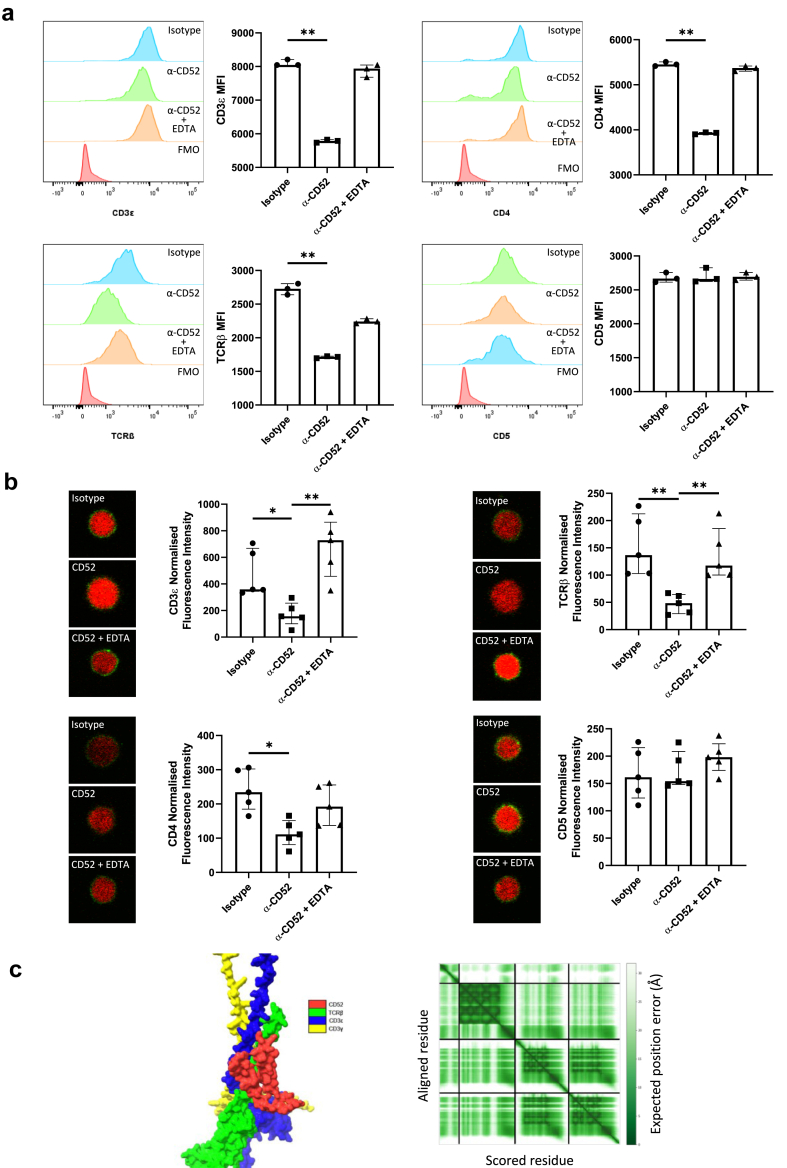
***Cis*-ligands of CD52 on CD4**^**+**^**T cells**. CD4^+^ T cells were magnetically isolated from PBMCs of patients with AD (n = 5) and stained with FITC-conjugated CD3ε, CD4, TCRβ or CD5 antibodies, as well as DABCYL-conjugated anti-CD52 antibody or isotype control, with or without the presence of 1 mM EDTA. **(a)** Fluorescence signal was measured with flow cytometry (n = 3). **(b)** Images of 20 cells from each sample (n = 5) were captured with fluorescence confocal microscopy (representative images shown in left panels), mean fluorescence intensity of FITC was measured. Green, FITC, membrane proteins; red, SYTO™ 59, nucleus. **(c)** AlphaFold Multimer-predicted structure of partial human TCR protein (TCRβ-CD3γ-CD3ε) and CD52. Top ranked model showing chains of TCRβ, CD3γ, CD3ε and CD52. CD52 interacts with TCRβ and CD3ε on their extracellular domain helices, but not with CD3γ (left panel). Predicted alignment error (PAE) for the top ranked model (right panel). Non-parametric statistical analysis (Kruskal–Wallis test followed by multiple comparisons with a two-stage step-up method of Benjamini, Krieger and Yekutieli) was used for multiple comparisons between three groups. Data are presented as median values with IQR. ∗p < 0.05 ∗∗p < 0.01 ∗∗∗p < 0.001.

CD4^+^ T cells isolated from 8 patients with AD were stained with a quencher (DABCYL)-conjugated anti-CD52 antibody or an isotype control. Subsequently, these cells were probed with FITC fluorescent-conjugated monoclonal antibodies specific to CD3ε, CD4, or TCRβ. The FRET pair of FITC and DABCYL has a Förster radius of 6.2 nm.^41^ The FRET effect, which resulted in dampening of the fluorescent signal, was detected using flow cytometry (n = 3) and confocal microscopy (n = 5). Anti-CD5 antibody was used as a negative control since CD5 was not identified as a potential ligand of CD52 through proximity labelling, and it is a highly expressed T cell marker but not part of the TCR complex. Flow cytometry results demonstrated that probing with CD3ε, CD4, or TCRβ antibodies significantly reduced the fluorescent signal of CD52, suggesting that cell membrane CD52 proteins on CD4^+^ T cells were within the 6.2 nm radius of CD3ε, CD4, or TCRβ (Fig. 4a). Treatment of cells with EDTA was insufficient to remove the interactions between these proteins and CD52. Despite having a similar abundance to CD3 and CD4 molecules on CD4^+^ T cells, CD5 probing did not affect the fluorescence intensity. Confocal microscopy, performed on a separate cohort of AD CD4^+^ T cells, corroborated these findings: anti-CD52 antibody was located within the proximity of CD3ε, CD4, and TCRβ antibodies, resulting in FRET effects (Fig. 4b). EDTA treatment interfered with protein interactions between CD3ε or TCRβ with CD52. Additionally, protein folding prediction analysis confirmed the interaction between CD52 and the extracellular domain of the TCRβ-CD3γ-CD3ε complex (Fig. 4c).

### *Cis*-interactions between CD52 and TCR interfere with TCR signalling and dampen antigen-dependent T cell activation

**Fig. 5:**
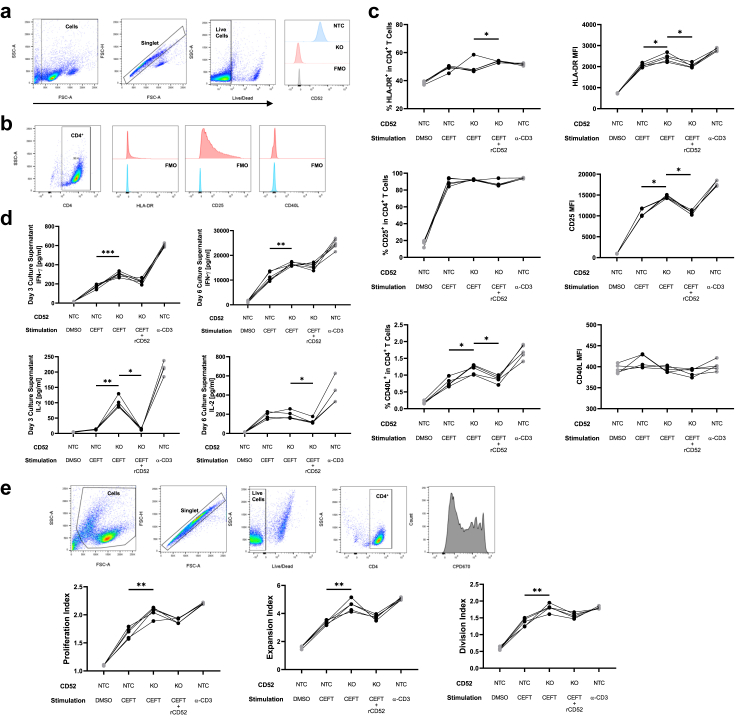
**Functional analysis of CD52 knockout (KO)**. CD4^+^ T cells were magnetically isolated from PBMCs of HC donors (n = 5) and underwent CRISPR KO of CD52. CD52^+^ cells were magnetically depleted in post-transfection cells to yield purified CD52 KO cells. **(a)** Representative flow cytometry histograms used to determine levels of membrane CD52 expression in CD52 KO or NTC cells, gating based on FMO controls. NTC or CD52 KO cells were co-cultured with MoDC and stimulated with CEFT peptide pool, anti-CD3 antibody or vehicle control. **(b)** Gating strategy and representative flow cytometry histograms used to determine levels of HLA-DR, CD25 and CD40L expression in CD4^+^ T cells. **(c)** Percentage expression of HLA-DR, CD25 and CD40L in CD4^+^ T cells (left panels), MFI of HLA-DR in HLA-DR^+^CD4^+^ T cells, MFI of CD25 in CD25^+^CD4^+^ T cells, MFI of CD40L in CD40L^+^CD4^+^ T cells (right panels) 3 days post-stimulation. **(d)** Concentrations of IFN-γ and IL-2 in cell culture supernatants 3 days (left panels) or 6 days (right panels) post-stimulation. **(e)** Gating strategy and representative flow cytometry histograms used to trace CD4^+^ T cell proliferation (top panels). Proliferation index, division index and expansion index of CD4^+^ T cells 6 days post-stimulation (bottom panels). Non-parametric statistical analysis (Friedman test followed by multiple comparisons with a two-stage step-up method of Benjamini, Krieger and Yekutieli) was used for multiple comparisons between the CEFT-stimulated groups. Data are presented as median values with IQR. ∗p < 0.05 ∗∗p < 0.01 ∗∗∗p < 0.001.

CD52 knockout in healthy primary CD4^+^ T cells was achieved using the CRISPR-Cas9 gene-editing system. Following gene editing, CD52-positive cells were depleted, thus enriching the population of CD52-negative cells. The depletion of CD52 protein expression was validated by flow cytometry (Fig. 5a). A polyclonal knockout was confirmed in the CD52-negative population using genomic DNA PCR (Supplemental Figure S3c). CD52 KO or non-targeted control (NTC) cells were co-cultured with allogeneic MoDCs and stimulated with the CEFT peptide pool. T cell activation marker (HLA-DR, CD25, and CD40L) expression was assessed in CD4^+^ T cells by flow cytometry on day 3 post-stimulation (Fig. 5b), and CD4^+^ T cell proliferation was assessed on day 6 post-stimulation. T cell activation-related pro-inflammatory cytokines, IFN-γ and IL-2, were measured in the culture supernatant. As expected, vehicle control only caused low levels of activation and proliferation in NTC cells; non-physiological activation using anti-CD3 antibodies to crosslink the TCR bypasses MHC recognition and served as the positive control, resulting the highest levels of activation and proliferation. CD52-KO cells exhibited increased expression of HLA-DR, CD25, and CD40L compared to that in NTC cells (Fig. 5c). These results were observed in CD4^+^ T cells from patients with AD. IFN-γ and IL-2 secretions, as well as HLA-DR and CD25 expressions were elevated after CD52 KO (Supplemental Figure S5).

Recombinant CD52 in the culture replenished the loss of soluble CD52 secretion from CD52 KO cells, but not membrane-bound CD52. Introducing rCD52 reversed the effects of CD52 KO, normalising the MFI of HLA-DR and CD25, and the percentage expression of CD40L (Fig. 5d). The same trend was observed for IFN-γ and IL-2 secretions. CD52 KO increased pro-inflammatory cytokine secretion upon CEFT stimulation compared to NTC cells. The proliferation index, division index and expansion index of CD4^+^ T cells 6 days post-stimulation were significantly increased compared to those of NTC cells (Fig. 5e). Nevertheless, the observed increase in cell proliferation was not mitigated by the addition of rCD52. Notably, the *in vitro* blockade of CD52 with an anti-CD52 antibody resulted in only a marginal increase in the activation and proliferation of NTC cells on day 3. However, this was followed by a marked decrease on day 6, accompanied by a high level of cell death (Supplemental Figure S4).

## Discussion

This study revealed an upregulation of CD52 expression in peripheral blood pan CD4^+^ T cells in patients with acute decompensation of cirrhosis, correlating with disease severity and 90-day mortality. We have identified that membrane-bound CD52 interacts in *cis*-membrane with the T-cell receptor complex, thereby interfering with TCR signalling and restricting antigen-specific T cell responses.

The expression level of Siglec-10, a recognised receptor of CD52, was elevated in CD4^+^ T cells from patients with AD. Siglec-10 is highly expressed in granulocytes, monocytes, and natural killer cells, but not in lymphocytes.^42^^,^^43^ Our findings indicate that typically low Siglec-10 expression levels were elevated in CD4^+^ T cells in AD, mirroring the trend observed with CD52. We initially hypothesised that this unusual elevation in Siglec-10 expression in CD4^+^ T cells might be an autocrine or paracrine effect of CD52 regulation. *Cis*-membrane interactions between lectins and their counter-receptors have been well reported and are known to be difficult to identify because of the weak nature of the lectin-glycan interaction.^4^, ^5^, ^6^^,^^44^^,^^45^ Quantitative proteomics following proximity labelling had enabled the unbiased discovery of neighbouring proteins to CD52 *in situ*, which could be potential *cis*-membrane ligands of CD52. However, Siglec-10 was not one of the seven proteins detected by proximity labelling, disproving that Siglec-10 is a CD52 *cis*-membrane ligand. The plate-bound avidity assay confirmed that within these seven proteins, CD3ε, CD4, and TCRβ showed high avidity towards CD52. As CD3ε and TCRβ are parts of the TCR protein complex, as well as CD4 being closely associated with the TCR as a co-receptor.^46^^,^^47^ We then hypothesised that CD52 might interact with the TCR complex. Moreover, proximity labelling identified non-membrane proteins directly associated with the downstream TCR signalling cascade: Lck and ZAP70, which further confirmed the spatial proximity of CD52 with the TCR complex.^48^^,^^49^ The detection of HRP and IGKV1D-43 in proteomic analysis, both components of the HRP-conjugated anti-CD52 antibody used in proximity labelling, served as a positive quality control for this methodology.

CD4, TCRβ, CD3ε, and CD3γ are at structural level closely positioned in the TCR complex.^46^ Therefore, although not discovered by proximity labelling, CD3γ was also tested in the avidity assay, yet CD3γ did not show high avidity towards CD52. IP-FCM under non-denatured conditions was used in attempt to isolate the TCR-CD52 complex. However, neither CD3ε, TCRβ, nor CD4 could be immunoprecipitated by CD52, implying that the TCR-CD52 affinity is weak or transient. Low affinity but high avidity protein–protein interactions are best known between glycans and glycan-binding proteins, where clustering of receptors and low-affinity ligands results in a high avidity overall interaction.^50^, ^51^, ^52^, ^53^, ^54^, ^55^ This interaction is weak and can only be observed *in situ*. Protein folding prediction indicated that CD52 interacts with the extracellular region of the TCR polymer, specifically within the constant domains of TCRβ and CD3ε, but not with CD3γ. This result is consistent with our experimental findings.

To investigate the function of *cis*-membrane interaction of CD52 with the TCR, we utilised CD52 KO primary CD4^+^ T cells in a physiological *in vitro* activation model with CEFT peptide-treated dendritic cells. Knockout of CD52 upregulated CD4^+^ T cell responses, as evidenced by increased cell activation, pro-inflammatory cytokine secretion, and proliferation, suggesting that CD52 had an inhibitory effect on antigen-dependent T cell activation. However, activating T cells unphysiologically through anti-CD3 antibodies, which crosslink CD3 and bypass the TCR recognition of MHC-II, could not be regulated by CD52. Recombinant CD52 replenished the loss of CD52 expression in KO cells and partially restored the inhibitory effects in terms of activation markers and short-term IL-2 secretion. Taken together with the protein interaction prediction analysis, this study shows that by interacting with the TCR constant regions in proximity to TCRβ and CD3ε, CD52 creates a steric hindrance to the recruitment of the T cell co-receptor CD4 and prevents MHC–II binding. This dynamic inhibition by CD52 may be a constant regulatory mechanism in CD4^+^ T cells, which modulates T cell activation by altering CD52 density. The upregulation of CD52 in CD4^+^ T cells in AD impairs antigen-specific T cell responses and thus potentially contributes to immunoparesis in AD, which leads to an increase in the risk of infection and mortality.

Alemtuzumab, a well-established humanised monoclonal antibody targeting CD52^25^^,^^26^ has been shown to have the potential to compete with the TCR complex for binding to CD52, thus removing the inhibition of CD52 towards T cell activation. Alemtuzumab has historically been used in the treatment of chronic lymphocytic leukaemia and is now used in multiple sclerosis, because of its ability to deplete CD52^+^ lymphocytes.^25^^,^^26^^,^^56^ In the treatment of multiple sclerosis however, autoimmune diseases, especially those affecting the thyroid, are common complications.^57^ The exact mechanism of this autoimmune assault is unclear, with the common hypothesis being the depletion of CD52-expressing regulatory T cells.^58^ The exact mechanism of depletion is not fully understood; however, it has been hypothesised to be driven by complement- and antibody-dependent cellular cytotoxicity.^59^^,^^60^ In this study, we attempted *in vitro* CD52 blockade with alemtuzumab; however, this antibody blockade did not enhance the activation and proliferation of CD52^+^CD4^+^ T cells. In contrast, alemtuzumab resulted in high levels of CD4^+^ cell death. This unfortunately suggests that although CD52 negatively regulates T cell activation, it may not be a suitable target for immune therapies using existing antibodies. To effectively target CD52, a modified antibody without adverse effects associated with lymphocyte depletion is needed.

Studies have demonstrated that CD52^high^CD4^+^ T cells exhibit suppressive effects on T cells. This suppression mechanism is thought to involve the release of soluble CD52 from cell membranes by phospholipase C, with soluble CD52 subsequently binding to the inhibitory receptor, Siglec-10.^28^ Ligation of Siglec-10 suppresses T cell activation downstream of the TCR by impairing the phosphorylation of TCR-associated kinases Lck and ZAP70. Here we propose an alternative suppressive mechanism upstream of the TCR, in which CD52 interferes with the TCR directly in the extracellular domain, providing TCR modulation independent of the formation of immune synapses. *Cis*-ligation on lymphocytes with functional consequences is a recognised mechanism of regulation.^4^ For instance, the CD48/58-CD2 interaction plays a role in recruiting Lck after TCR activation, and CD22 interacts with the *cis*-ligand Siglec-2, which inhibits B cell receptor signalling.^5^^,^^6^

We reported the upregulation of CD52 in CD4^+^ T cells in a cohort of 28 patients with AD, with 71% of the aetiology being alcohol-related liver disease. This observation requires further validation in a larger patient cohort, with more representation of subgroups characteristics, such as age and aetiologies. Mechanisms responsible for the induction of this phenotype in relation to cirrhosis progression and precipitating events for decompensation require further investigation. Although this study focused on circulating lymphocytes in acute decompensation of cirrhosis, a single-cell RNA sequencing atlas of cirrhotic and uninjured livers revealed upregulation of *CD52* gene in CD4^+^ T cells in cirrhotic livers.^61^ The role of CD52 in the intrahepatic microenvironment remains to be elucidated. Further exploration in diverse disease cohorts such as patients with sepsis without underlaying cirrhosis or liver disease is warranted to further determine the potential role of liver injury in the development of this phenotype and dissect the role of this population in infection. Although available, murine models do not fully recapitulate human liver disease,^62^^,^^63^ but they might provide further insights into potential mechanisms of disease pathogenesis that might account for the observed upregulation of CD52.

Various reports have previously identified mediators implicated in adaptive immune alterations that contribute to immune suppression in AD. Here, we report ubiquitous interference with TCR engagement, a prerequisite step for the initiation of T cell activation. The upregulated cell membrane CD52 demonstrated *cis-*interaction with the TCR on CD4^+^ T cells, resulting in the suppression of antigen-specific activation through the hindrance of TCR-MHC binding. Taken together, our study indicates that more than one threshold of activation must be surpassed to overcome the attenuated responses to infections observed in AD.

## Contributors

WK initiated the study. TL coordinated the study. GW, YL, MT contributed to the conceptualisation and approach to the study. TL, GW, CG, TB, WK contributed to data curation, analyses, investigation, methodology and interpretation of data. ET, TB, FT, MM provided and contributed to supporting methodology, supporting resources and material. TL, GW, WK have accessed and verified the data. TL wrote the original draft. TL, GW, CG, FT, TB, ET, YL, MM, WK, MT contributed to the reviewing and editing of the original draft. WK, MT acquired funding. All authors read and approved the final version of the manuscript.

## Data sharing statement

All data relevant to the study are included in the article or uploaded as supplementary information.

## Declaration of interests

Authors disclose no conflicts.

## Appendix A Supplementary data

Supplementary Figures **Figure S1. CD52 and Siglec-10 expressions in T cell populations in patients with acute decompensation of cirrhosis (AD, n=28), chronic decompensation of cirrhosis (CD, n=21) and healthy controls (HC, n=17). (a)** Percentage of CD52 expressing cells in CD4^+^ T cells, CD8^+^ T cells or Tregs (top panels). Median fluorescence intensity (MFI) of CD52 in CD52^+^CD4^+^ T cells, CD52^+^CD8^+^ T cells or CD52^+^ Tregs (bottom panels). **(b)** Percentage of Siglec-10 expressing cells in CD4^+^ T cells, CD8^+^ T cells or Tregs. **(c)** Gating strategy and representative flow cytometry histograms used to determine levels of CD52 and Siglec-10 in CD4^+^ T cells, CD8^+^ T cells, CD4^+^CD25^+^CD127^-^ Tregs and CD4^+^HLA-G^+^ suppressive T cells. All gating based on fluorescence-minus-one (FMO) controls. **(d)** Percentage CD52 expressing cells in CD4^+^HLA-G^+^ suppressive T cells and MFI of CD52 in CD52^+^CD4^+^HLA-G^+^ suppressive T cells (left panels). Percentage Siglec-10 expressing cells in CD4^+^HLA-G^+^ suppressive T cells and MFI of Siglec-10 in Siglec-10^+^CD4^+^HLA-G^+^ suppressive T cells (right panels). **(e)** Distribution of CD52 MFI in CD4^+^ T cells in patients with AD who had culture-positive infections at admission (n = 10) and those who were culture-negative (n=18). **(f)** Correlation between CD52 MFI in CD4^+^ T cells and serum CRP levels in patients with AD. **(g)** Correlation between CD52 MFI in CD4^+^ T cells and age in AD (left panel), CD (middle panel), and HCs (right panel). **(h)** CD52 MFI in CD4^+^ T cells in patients with AD who were below or above median age of the cohort (41.5-year-old). **(i)** Age sensitivity test of CD52 MFI in CD4^+^ T cells. By removing 10 oldest patients with AD or CD from comparison, no significant difference in age among AD, CD and HC cohorts was achieved (upper panel). CD52 MFI in CD4^+^ T cells in patients with AD remained significantly elevated compared to patients with CD or HCs (lower panel). Non-parametric statistical analysis was used (Mann-Whitney test for two group comparison, Kruskal-Wallis test for unpaired data, or Friedman test for paired data, followed by multiple comparisons with two-stage step-up method of Benjamini, Krieger and Yekutieli for multiple comparisons between more than two groups). Data are presented as median values with IQR. Correlation coefficient (r) and p value were tested using Spearman’s correlation test. ∗p < 0.05 ∗∗p < 0.01 ∗∗∗p < 0.001 **Figure S2. (a)** Table showing not significant changed membrane proteins identified by proximity labelling. **(b)** Volcano plot showing cytoplasmic proteins identified in quantitative proteomics. Proteins ZAP70, LCK, HRP and IGKV1D-43 are highlighted in red. **Figure S3. (a)** Dose response of avidity between CD52 and potential ligands. Fluorescence from immobilised Alexa Fluor 488-conjugated recombinant CD52 proteins was measured after probing with DABCYL-conjugated recombinant Siglec-10, TCRβ, CD3ε, CD4, human IgG from 0.125μM to 1M. Data are presented as median values with IQR. **(b)** Representative agarose gel electrophoresis of CD52 gene PCR amplicons. PCR performed on genome DNA of CD52 knockout (KO) and non-targeted control (NTC) primary CD4^+^ T cells from two donors. NTC showed 3391 bp bands of wild type CD52 gene. KO showed polyclonal knockout of CD52 gene. M, DNA marker. **Figure S4. Functional analysis of CD52 knockout (KO) and CD52 blockade in non-targeted control (NTC).** CD4^+^ T cells were co-cultured with monocyte-derived dendritic cells and stimulated with CEFT peptide pool, with or without 10 μg/ml CD52 monoclonal antibodies (alemtuzumab biosimilar). **(a)** Percentage expression of HLA-DR, CD25 and CD40L in CD4^+^ T cells (top panels), median fluorescence intensity (MFI) of HLA-DR in HLA-DR^+^CD4^+^ T cells, MFI of CD25 in CD25^+^CD4^+^ T cells, MFI of CD40L in CD40L^+^CD4^+^ T cells (bottom panels) 3 days post-stimulation. **(b)** Proliferation index, division index and expansion index of CD4^+^ T cells 6 days post-stimulation. **(c)** Representative flow cytometry histograms showing CD4^+^ T cell viability 3 days or 6 days post-stimulation. Non-parametric statistical analysis (Friedman test followed by multiple comparisons with two-stage step-up method of Benjamini, Krieger and Yekutieli) was used for multiple comparisons. Data are presented as median values with IQR. ∗p < 0.05 ∗∗p < 0.01 **Figure S5. Functional analysis of CD52 knockout (KO) in AD CD4**^**+**^**T cells.** CD4^+^ T cells were magnetically isolated from PBMCs of patients with AD (n = 5) and underwent KO of CD52. CD52^+^ cells were magnetically depleted in post-transfection cells to yield purified CD52 KO cells. **(a)** Gating strategy and representative flow cytometry histograms used to determine levels of HLA-DR, CD25 and CD40L expression in CD4^+^ T cells. **(b)** Percentage expression of HLA-DR, CD25 and CD40L in CD4^+^ T cells (left panels), MFI of HLA-DR in HLA-DR^+^CD4^+^ T cells, MFI of CD25 in CD25^+^CD4^+^ T cells, MFI of CD40L in CD40L^+^CD4^+^ T cells (right panels) 3 days post-stimulation. **(c)** Concentrations of IFN-γ (left panel) and IL-2 (right panel) in cell culture supernatants 3 days post-stimulation. Repeated-measures one-way ANOVA, with Geisser-Greenhouse correction, followed by multiple comparisons with a two-stage step-up method of Benjamini, Krieger and Yekutieli was used for multiple comparisons. ∗p < 0.05 ∗∗p < 0.01
