# Supplementary material for: Cis-interaction between CD52 and T cell receptor complex interferes with CD4+ T cell activation in acute decompensation of cirrhosis
Source: eBioMedicine. 2024 Sep 13;108:105336. doi: 10.1016/j.ebiom.2024.105336 (PMC11418137; doi:10.1016/j.ebiom.2024.105336)

Supplementary Figure S1

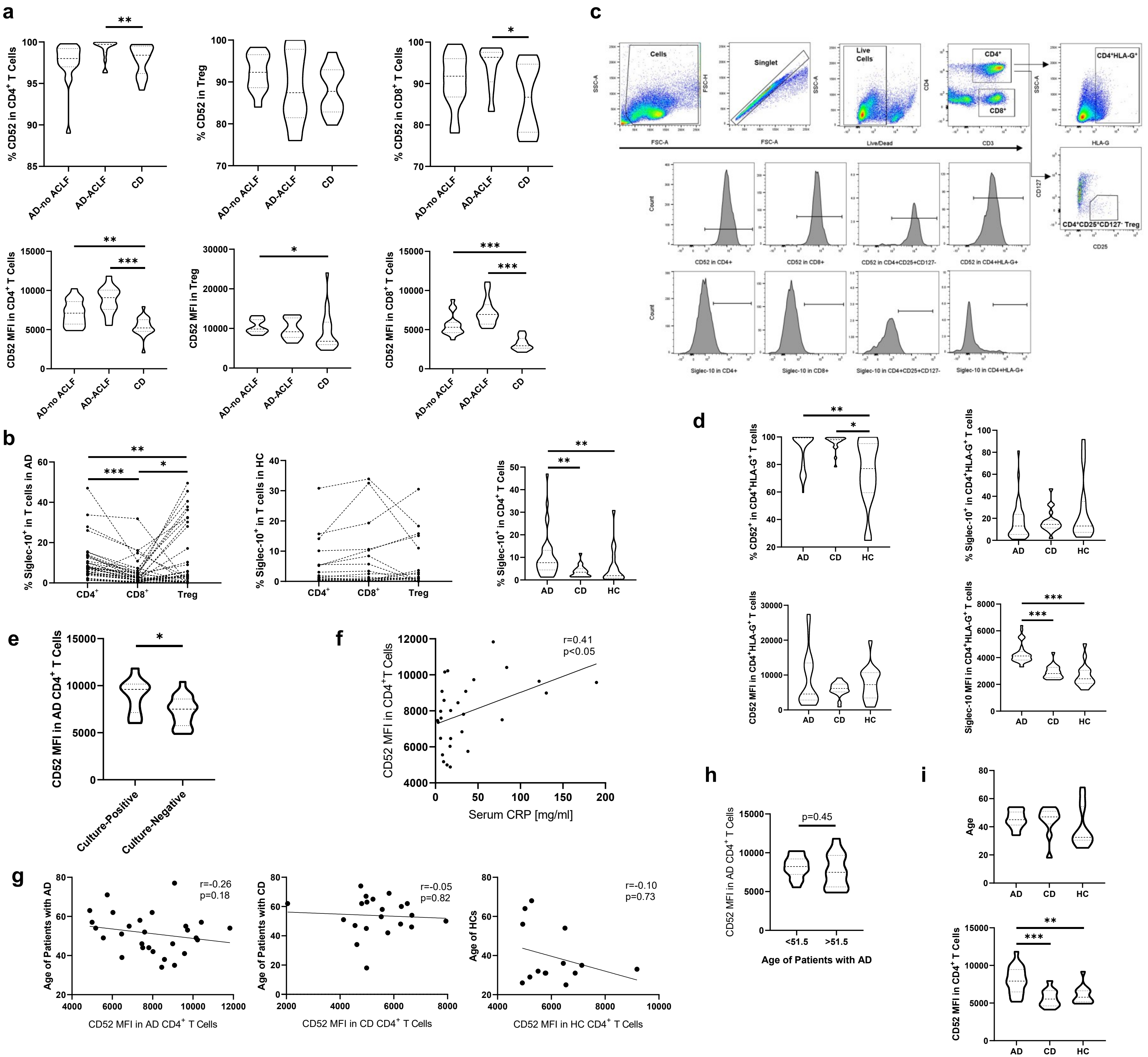

Supplementary Figure S2

a

Not significantly changed membrane proteins identified by proximity labelling					
**Gene name**	**Log2 fold change**	**P value**	**Gene name**	**Log2 fold change**	**P value**
HLA-DRB1	7.033	0.001253	GNB1/2	5.465	0.01111
ESYT2	6.244	0.001296	RASAL3	5.511	0.01994
SEC61A1/2	5.159	0.001315	RAC1/2	2.105	0.02119
GNAI3	5.847	0.001470	ITGAL	5.064	0.02509
EEF1A1	1.824	0.002313	HLA-B	7.463	0.02939
GNAI2	4.901	0.002535	HLA-A	6.548	0.03782
SLC3A2	6.054	0.002580	VDAC2	2.443	0.03951
LCP1	5.385	0.003080	SAMHD1	3.730	0.06338
CD6	6.547	0.003368	FLNA	2.121	0.06469
B2M	6.509	0.003513	CORO1A	5.645	0.07761
ENO1	5.114	0.003814	MYH9	1.812	0.09301
DEF6	3.574	0.004079	INADL	-1.701	0.1067
CSK	4.967	0.004297	GIMAP5	5.935	0.1076
PTPRC	4.530	0.004297	HSPA8	3.738	0.1242
CD5	4.975	0.004426	GAPDH	4.315	0.1256
GNA13	4.663	0.005176	MYO1G	2.398	0.2299
ITGA6	5.570	0.009466			

b

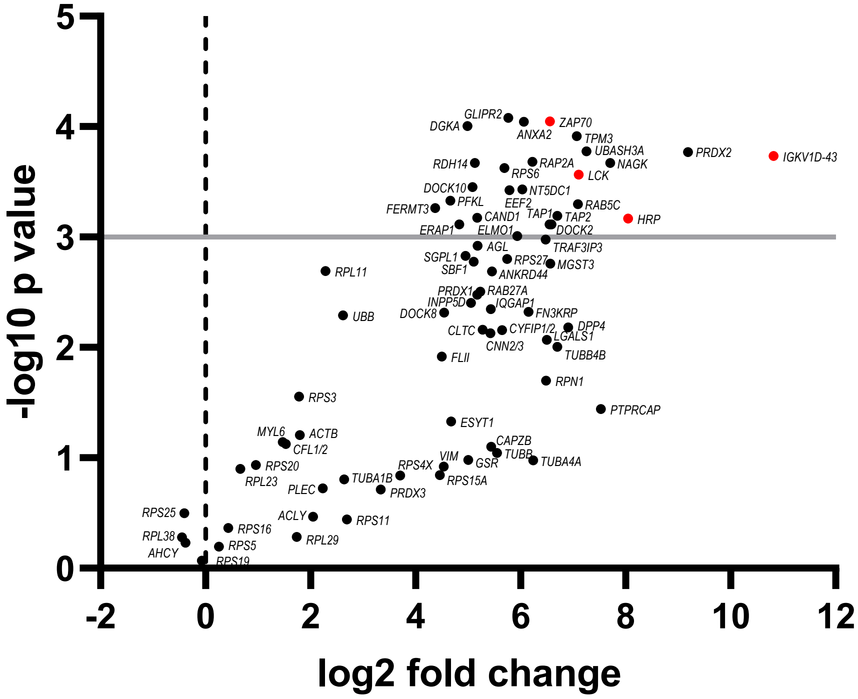

Supplementary Figure S3

a

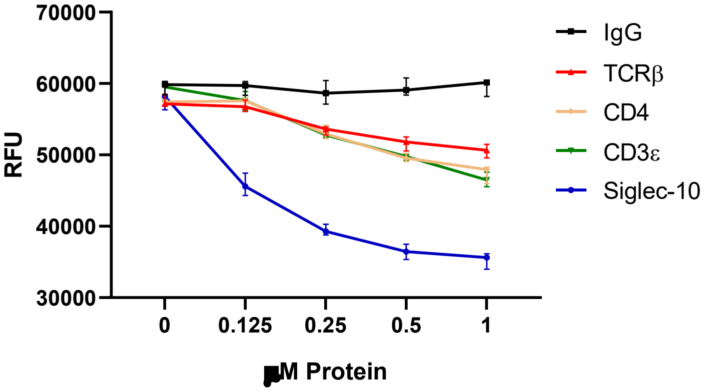

b

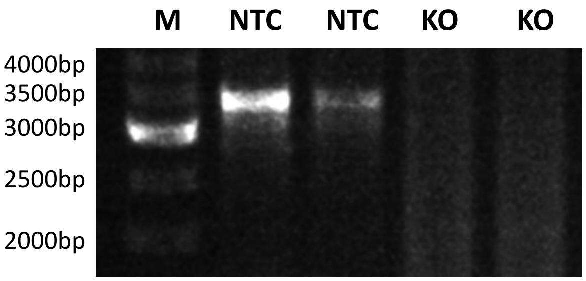

Supplementary Figure S4

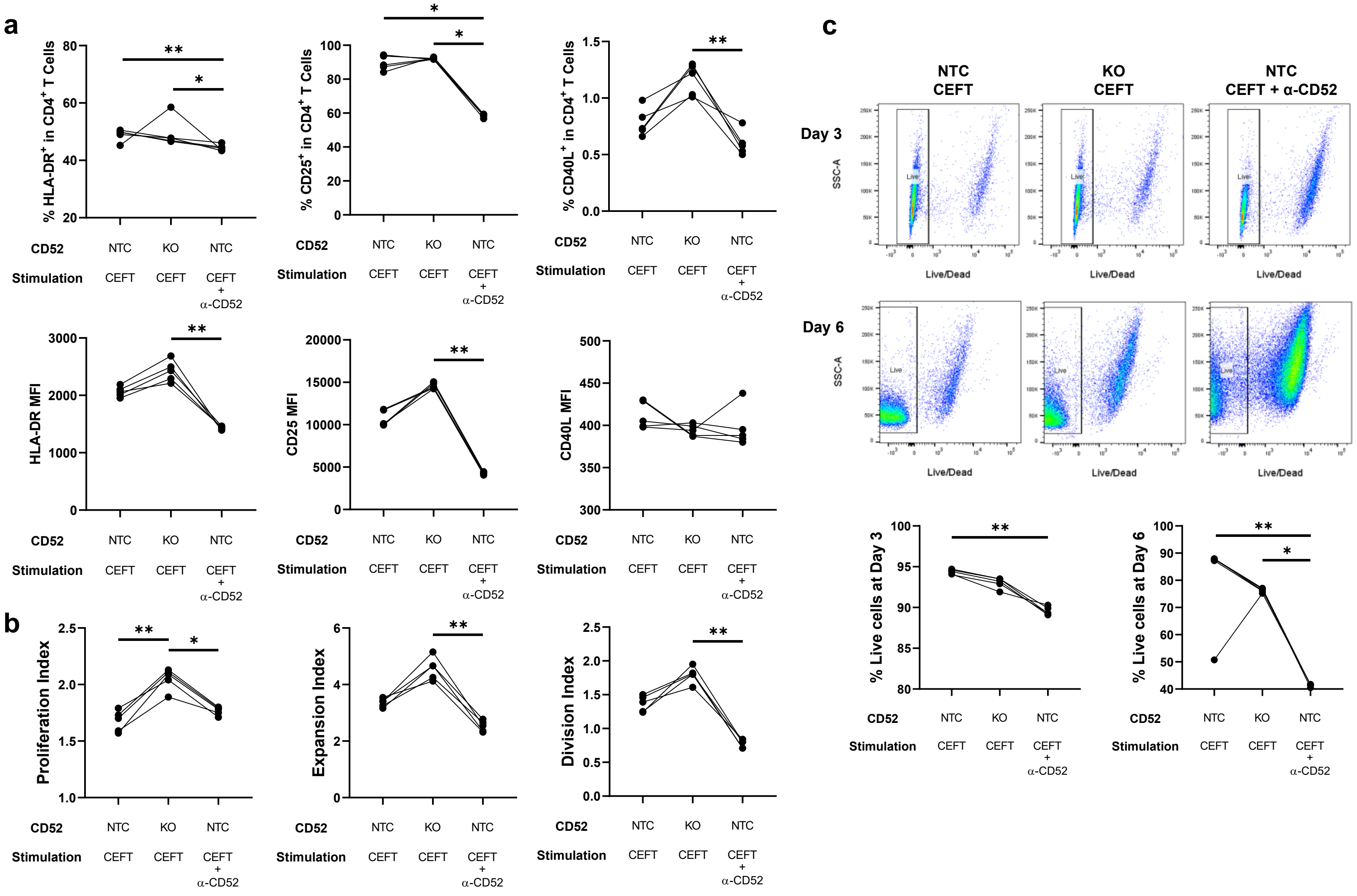

Supplementary Figure S5

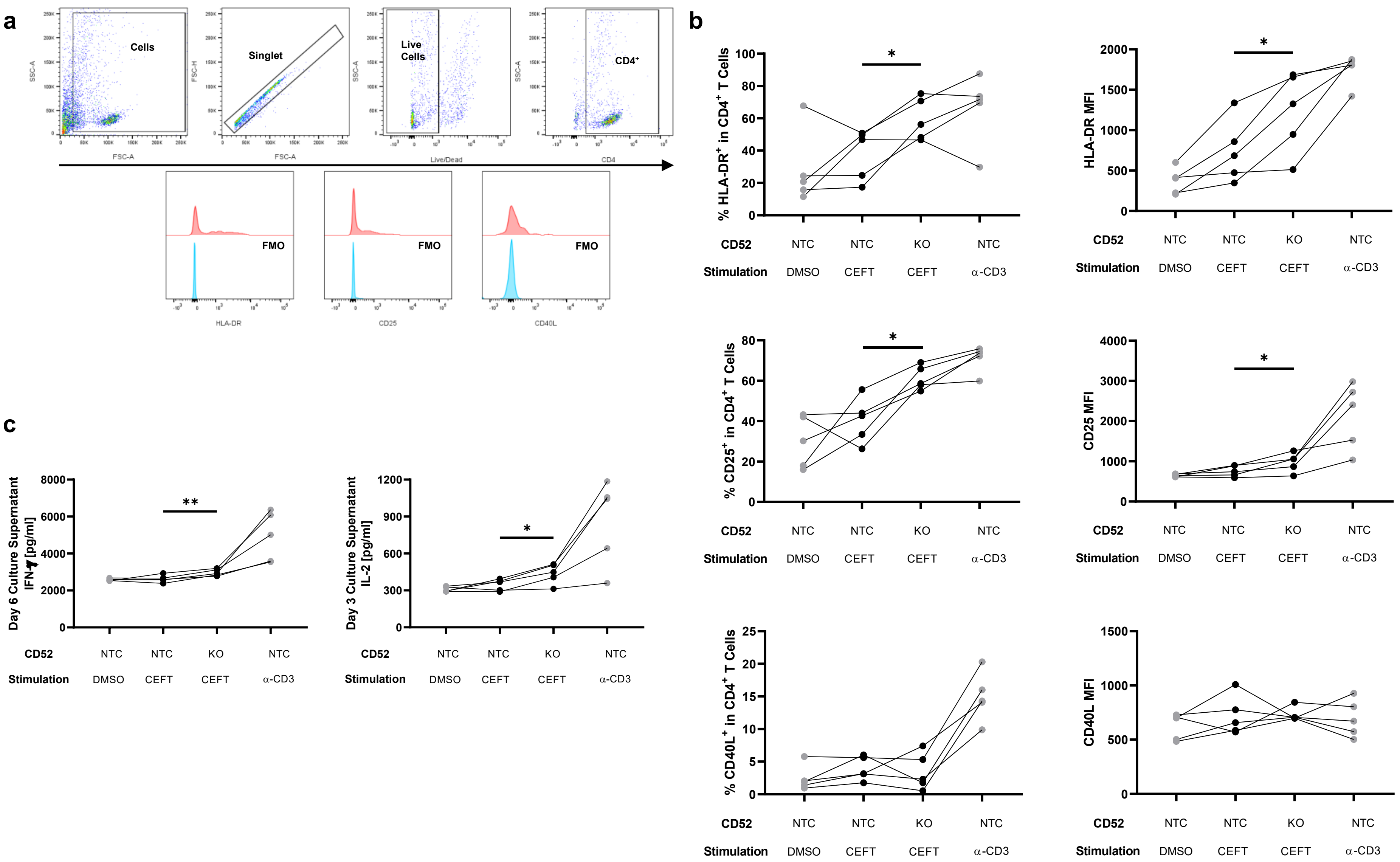

Supplement: Supplementary Figures — Figure S1. CD52 and Siglec-10 expressions in T cell populations in patients with acute decompensation of cirrhosis (AD, n=28), chronic decompensation of cirrhosis (CD, n=21) and healthy controls (HC, n=17). (a) Percentage of CD52 expressing cells in CD4+ T cells, CD8+ T cells or Tregs (top panels). Median fluorescence intensity (MFI) of CD52 in CD52+CD4+ T cells, CD52+CD8+ T cells or CD52+ Tregs (bottom panels). (b) Percentage of Siglec-10 expressing cells in CD4+ T cells, CD8+ T cells or Tregs. (c) Gating strategy and representative flow cytometry histograms used to determine levels of CD52 and Siglec-10 in CD4+ T cells, CD8+ T cells, CD4+CD25+CD127- Tregs and CD4+HLA-G+ suppressive T cells. All gating based on fluorescence-minus-one (FMO) controls. (d) Percentage CD52 expressing cells in CD4+HLA-G+ suppressive T cells and MFI of CD52 in CD52+CD4+HLA-G+ suppressive T cells (left panels). Percentage Siglec-10 expressing cells in CD4+HLA-G+ suppressive T cells and MFI of Siglec-10 in Siglec-10+CD4+HLA-G+ suppressive T cells (right panels). (e) Distribution of CD52 MFI in CD4+ T cells in patients with AD who had culture-positive infections at admission (n = 10) and those who were culture-negative (n=18). (f) Correlation between CD52 MFI in CD4+ T cells and serum CRP levels in patients with AD. (g) Correlation between CD52 MFI in CD4+ T cells and age in AD (left panel), CD (middle panel), and HCs (right panel). (h) CD52 MFI in CD4+ T cells in patients with AD who were below or above median age of the cohort (41.5-year-old). (i) Age sensitivity test of CD52 MFI in CD4+ T cells. By removing 10 oldest patients with AD or CD from comparison, no significant difference in age among AD, CD and HC cohorts was achieved (upper panel). CD52 MFI in CD4+ T cells in patients with AD remained significantly elevated compared to patients with CD or HCs (lower panel). Non-parametric statistical analysis was used (Mann-Whitney test for two group comparison, Kruskal-Wallis test for unpair [file mmc1.pdf]
