# Supplementary material for: Cis-interaction between CD52 and T cell receptor complex interferes with CD4+ T cell activation in acute decompensation of cirrhosis
Source: eBioMedicine. 2024 Sep 13;108:105336. doi: 10.1016/j.ebiom.2024.105336 (PMC11418137; doi:10.1016/j.ebiom.2024.105336)
Supplement: Supplementary Material [file mmc2.pdf]

Supplementary Materials for

Cis-interaction between CD52 and T cell receptor complex interferes with $CD4^+$ T cell activation in acute decompensation of cirrhosis

Tong Liu[1], Gang Wu[2], Cathrin L.C. Gudd[1], Francesca M. Trovato[3], Thomas Barbera[1], Yan Liu[4], Evangelos Triantafyllou[1], Mark J.W. McPhail[3], Mark R. Thursz [1¶] and Wafa Khamri[1¶]

[(1)]Section of Hepatology & Gastroenterology, Division of Digestive Diseases, Department of Metabolism, Digestion & Reproduction, Imperial College London, London, United Kingdom; [(2)]Department of Life Sciences, Imperial College London, London, United Kingdom; [(3)]Department of Inflammation Biology, Institute of Liver Studies, King's College London, London, United Kingdom; [(4)]Glycosciences Laboratory, Department of Metabolism, Digestion & Reproduction, Imperial College London, London, United Kingdom;

*** Corresponding author:**
Dr Wafa Khamri
Imperial College, Liver Immunology Laboratory
Division of Digestive Disease
Department of Metabolism, Digestion & Reproduction
10th Floor QEQM Wing, St Mary's Campus
South Warf Road
W2 1NY London, UK
Tel: +44 (0) 203 3126454
Email: w.khamri@imperial.ac.uk

¶ Authors share last co-authorship

Supplementary Material and Methods

Patient characteristics

Informed consent was obtained from patients or if the patient lacked capacity, assent was sought from the next of kin. All patients with a diagnosis of cirrhosis admitted to hospital were screened on admission. Inclusion criteria: clinical and/or biochemical and/or radiology and/or histological diagnosis of cirrhosis, hospital admission with complication of cirrhosis including alcoholic hepatitis, sepsis, variceal haemorrhage, ascites, renal dysfunction, and commencement of antimicrobial therapy. Exclusion criteria were the following: patients younger than 18 years; pregnancy; malignancy; active viral infection (hepatitis A/B/C/E viruses or human immunodeficiency virus); immunosuppression (excluding low dose steroids or steroid sparing agents for autoimmune hepatitis treatment - < 20 mg or equivalent of prednisolone); type 1 diabetes mellitus; inflammatory bowel disease; coeliac disease.

Peripheral blood mononuclear cell isolation and flow cytometry

Peripheral blood mononuclear cells (PBMCs) were isolated from lithium heparin-anticoagulated whole blood through Ficoll-paque™ Plus (GE Healthcare Bio-Sciences AB, Sweden) density-gradient centrifugation, cryopreserved and stored at -80°C. Following fixable viability dye (FVD) staining (Thermo Fisher Scientific, MA, USA), cells were surface stained using fluorochrome-labelled mouse anti-human monoclonal antibodies (**Supplementary Table S1**). Antibodies validated by manufacturers by flow cytometry. Fluorescence minus one (FMO) were used as controls for all gating. Data was acquired on the BD LSRFortessa™ flow cytometer using BD

FACSDiva™ software (Becton Dickinson Ltd, Oxford, UK) and analyses were performed using FlowJo™ software V10.4 (Becton Dickinson Ltd).

Cell sorting and transcriptomics profiling

PBMCs were surface stained with FVD, CD3, CD4, CD8, CD25 and CD127 antibodies (as described above). Flow-based cell sorting was performed on BD FACSAria-III™ cell sorter using BD FACSDiva™ software (Becton Dickinson Ltd). First, $CD25^{+}CD127^{-}$ regulatory T cells (Treg) were isolated. Then, effector T cells were sorted from $CD25^{low}CD127^{high}$ fraction after HLA-G depletion. The sorted cells were lysed using RLT lysis buffer (Qiagen, Manchester, UK) supplemented with 1% (v/v) β- mercaptoethanol and stored at -80°C. RNeasy mini kit (Qiagen) was used to extract RNA from cell lysate. This was followed by cDNA synthesis with Bio-rad iScript cDNA synthesis kit (Bio-Rad, Hertfordshire, United Kingdom), according to the manufacturers' instructions. The NanoString nCounter GX Human Immunology V2 assay (NanoString Technologies, Seattle, Washington, USA) was performed at the UCL NanoString Facility (University College London, UK). Analyses of 770 immune-related genes were performed. Gene expression was reported as log2 fold change of detected mRNA expression levels, normalised to baseline values of effector T cells. Statistical significance was considered for $p<0.05$ and a log2 fold change of 1.5 times increase or decrease. Data analysed using the NanoString nSolver™ Analysis Software V4.0 with NanoString Advanced Analysis Module 2.0 plugin (NanoString Technologies).

Proximity labelling and quantitative proteomics

For CD52 proximity labelling, 2×10^{6} magnetic beads-isolated AD $CD4^{+}$ T cells

(n=4) were stained with 30 µg/ml horseradish peroxidase (HRP)-conjugated mouse anti-human CD52 monoclonal antibody (RRID: AB_3402603) or mouse IgG1 isotype (RRID: AB_3242323) (R&D Systems, Abingdon, UK) at 4°C for 20 min. Stained cells were incubated with 95µM tyramide-SS-biotin (Iris Biotech, Germany) and 0.01% (v/v) hydrogen peroxide (Sigma-Aldrich, Dorset, UK) on ice for 2 min. Labelled cells were washed 2 times with 3 mL 100 U/mL bovine liver catalase (Sigma-Aldrich) and 2 times with 3mL PBS (Thermo Fisher Scientific). Validation of successful labelling was confirmed by flow cytometry using allophycocyanin (APC)-conjugated streptavidin (Thermo Fisher Scientific). Cells were lysed in 20mM Tris-HCl, 5mM EDTA, 150mM NaCl, 1% (v/v) Triton X-100, 0.1M sodium thiocyanate (Sigma-Aldrich), pH 8.0 supplemented with protease inhibitor cocktail (Roche, Boehringer, UK). Streptavidin dynabeads (Thermo Fisher Scientific) were incubated with cell lysate at 4°C overnight. The beads were then washed sequentially in 10mM Tris–Cl, 1% (v/v) Triton X-100, 1mM EDTA, 0.5% (w/v) SDS, 500mM NaCl, 0.1M sodium thiocyanate, pH 7.4, followed by 10mM Tris-HCl, 1% (v/v) Triton X-100, 1mM EDTA, 0.5% (w/v) SDS, 0.1M sodium thiocyanate, pH 7.4 and PBS at room temperature for 10 min each. The bound proteins were eluted using 50mM Tris, 5mM EDTA, 150mM NaCl, 1% SDS, 13mM TCEP (Sigma-Aldrich), pH 8.0 at room temperature for 10 min. Purified proteins were carboxymethylated by iodoacetamide, cleaned by SP3 paramagnetic beads beads, and digested by trypsin (Promega, Wisconsin, USA).[36] Peptides were submitted to Orbitrap Q Exactive HF (Thermo Fisher Scientific) for proteomic analysis. The proteomic raw data were imported to MaxQuant Version 2.0.3.0 to search protein FASTA files in Uniprot database.[64] The label-free

quantification intensities of proteins were used for downstream analysis by Perseus Version 1.6.0.7 and R Version 3.6.3.[65] Gene Ontology Cellular Component (GOCC) analysis was based on the reviewed human protein entries in Uniprot database. Membrane proteins identified were visualised by volcanic plot.

Fluorochrome labelling of proteins

Recombinant human CD52 protein (R&D Systems) was conjugated with Alexa Fluor 488 dye using Thermo Fisher Alexa Fluor labelling kit (Thermo Fisher Scientific): recombinant CD52 were reconstituted at 1 mg/ml in pH 8.3 0.1M sodium bicarbonate solution and labelled with Alexa Fluor 488-succinimidyl ester at 1:1 ratio at room temperature for 1 hour. Unbound dyes were separated from conjugated proteins with resin purification column. Other recombinant proteins were conjugated with 4-((4-(dimethylamino)phenyl)azo)benzoic acid (DABCYL) using DABCYL-succinimidyl ester (DABCYL-SE) (Sigma-Aldrich): proteins were reconstituted at 2 mg/ml in pH 8.3 0.1M sodium bicarbonate solution and labelled with DABCYL-SE at 1:1 ratio a room temperature for 1 hour. Reaction was terminated by 1.5M hydroxylamine pH 8.5 (Sigma-Aldrich). Unbound DABCYL were separated from conjugated proteins with Nanosep 10K Omega centrifugal membrane (Pall Corporation, Portsmouth, UK).

Fluorescence confocal microscopy

Stained cells were resuspended in Flow Cytometry Staining Buffer (Thermo Fisher Scientific) at 10^5 cell/ml and placed on chamber slides (Corning, NY, USA). The slides were imaged with inverted Leica TCS SP5 confocal microscope with Leica DM6000 confocal fixed stage (Leica Biosystems,

Germany). Five field-of-views were captured under 100x microscope objective lens. Images of 20 cells were processed and analysed on ImageJ V1.53 software (National Institutes of Health, MD, USA), average cell membrane fluorescein isothiocyanate (FITC) signal normalised to nucleus SYTO™ 59 signal of the 20 cells were presented in histograms.

CD52 knockout

To prepare guide RNA (gRNA), 100μM chemically synthesised trans-activating RNA (tracrRNA)and CRISPR RNA (crRNA) (Integrated DNA Technologies, IA, USA) were mixed at 1:1 ratio and annealed at 95°C for 5 min. The sequences of the crRNAs are provided in **supplementary table S3**. Ribonucleoprotein complexes were assembled by incubating equal volume of 44μM gRNA with 36μM Alt-R™ S.p. Cas9 V3 nuclease (Integrated DNA Technologies) at room temperature for 20 min. Magnetic beads-isolated CD4^{+} T cells from HC were activated with dynabead T cell activator (Thermo Fisher Scientific) at 2:1 cells-to-beads ratio for 2 days. Ribonucleoprotein at 20μM was delivered to 5x10^{5} cells, along with 10.8μM electroporation enhancer (Integrated DNA Technologies), using Neon™ transfection system (Thermo Fisher Scientific). The Neon™ transfection system delivered 3 pulses of 10 ms 1600 V electrical shocks. Transfected cells were culture in RPMI-1640 medium supplemented with 10% (v/v) foetal bovine serum (Thermo Fisher Scientific) for 2 days. Then, transfected cells were surface stained with FITC-conjugated CD52 monoclonal antibody, and CD52^{+} cells were depleted using positive magnetic beads selection with anti-FITC microbeads (Miltenyi Biotec), CD52^{-} cells were collected as CD52 KO cells for downstream experiments. CD52 KO was validated at protein and genomic levels. Cell surface CD52 expression was

detected by flow cytometry using Super Bright 702-conjugated CD52 monoclonal antibody (Thermo Fisher Scientific). Genomic DNA was extracted using QIAamp DNA Mini Kit (Qiagen, Manchester, UK), and full length *CD52* exon was amplified using flanking primers: forward 5'-CACCAACCCTTCCACA ATCTA-3'; reverse, 5'-GGCATCTCTAGTACCATCATTCC-3' (Integrated DNA Technologies); yielding a 3391 bp product. Amplicons were visualised on agarose gel after electrophoresis.

Monocyte-derived dendritic cells

$CD14^+$ monocytes were magnetic beads-isolated from HC PBMC by positive selection with human CD14 microbeads (Miltenyi Biotec) and cultured in TexMACS medium (Miltenyi Biotec) supplemented with 10% human male AB serum (Thermo Fisher Scientific) at a density of 10^6 cells/ml. Monocyte-derived dendritic cells (MoDC) were differentiated with 100 ng/ml recombinant human GM-CSF and 50 ng/ml recombinant human IL-4 (Miltenyi Biotec) for 7 days.

Supplementary Tables

Table S1. Antibodies used for flow cytometry phenotyping				
**Laser-Bandpass filter**	**Antigen**	**Fluorochrome**	**Clone**	**RRID**
Violet 405-450/50	CD3 [1]	eFluor450	SK7	AB_11217677
Violet 405-525/50	CD4 [2]	BV510	SK3	AB_2870492
Violet 405-710/50	CD52 [1]	Super Bright 702	CF1D12	AB_2762576
Violet 405-780/60	Siglec-10 [2]	BV786	5G6	AB_2744259
Blue 488-530/30	HLA-G [1]	FITC	MEM-G/9	AB_1076722
Blue 488-610/20	CD25 [2]	PE-CF594	M-A251	AB_2737636
Blue 488-780/60	CD127 [2]	PE-Cy7	HIL-7R-M21	AB_2033938
Red 640-670/14	CD8 [1]	APC	RPA-T8	AB_469338
Red 640-780/60	Fixable Viability Dye (FVD) [1]	eFluor780	N/A	N/A

1 Thermo Fisher Scientific, MA, USA
2 Becton Dickinson Ltd, Oxford, UK

Table S2. Recombinant protein sequences			
**Protein**	**Sequence**	**C-terminus modification**	**Expression platform**
CD52	MKRFLFLLLTISLLVMVQIQTGLSGQNDTSQTSSPS	IgG1 Fc	HEK293
TCRβ1	DLNKVFPPEVAVFEPSEAEISHTQKATLVCLATGFFPDHVELSWWVNGKEVHSGVST DPQPLKEQPALNDSRYCLSSRLRVSATFWQNPRNHFRCQVQFYGLSENDEWTQDR AKPVTQIVSAEAWGRAD	Myc	*E. coli*
CD3ε	DGNEEMGGITQTPYKVSISGTTVILTCPQYPGSEILWQHNDKNIGGDEDDKNIGSDE DHLSLKEFSELEQSGYYVCYPRGSKPEDANFYLYLRARVCENCMEMDVMSVATIVIV DICITGGLLLLVYYWSKNRKAKAKPVTRGAGAGGRQRGQNKERPPPVPNPDYEPIRK GQRDLYSGLNQRRI	Myc	*E. coli*
CD3γ	QSIKGNHLVKVYDYQEDGSVLLTCDAEAKNITWFKDGKMIGFLTEDKKKWNLGSNA KDPRGMYQCKGSQNKSKPLQVYYRMCQNCIELNAATIS	Myc	*E. coli*
CD43	STTAVQTPTSGEPLVSTSEPLSSKMYTTSITSDPKADSTGDQTSALPPSTSINEGSPLWT SIGASTGSPLPEPTTYQEVSIKMSSVPQETPHATSHPAVPITANSLGSHTVTGGTITTN SPETSSRTSGAPVTTAASSLETSRGTSGPPLTMATVSLETSKGTSGPPVTMATDSLETS TGTTGPPVTMTTGSLEPSSGASGPQVSSVKLSTMMSPTTSTNASTVPFRNPDENSR	Myc	*E. coli*
CD44	QIDLNITCRFAGVFHVEKNGRYSISRTEAADLCKAFNSTLPTMAQMEKALSIGFETCRY GFIEGHVVIPRIHPNSICAANNTGVYILTSNTSQYDTYCFNASAPPEEDCTSVTDLPNA FDGPITITIVNRDGTRYVQKGEYRTNPEDIYPSNPTDDDVSSGSSSERSSTSGGYIFYTF STVHPIPDEDSPWITDSTDRIP	IgG1 Fc	HEK293
CD48	QGHLVHMTVVSGSNVTLNISESLPENYKQLTWFYTFDQKIVEWDSRKSKYFESKFKG RVRLDPQSGALYISKVQKEDNSTYIMRVLKKTGNEQEWKIKLQVLDPVPKPVIKIEKIE DMDDNCYLKLSCVIPGESVNYTWYGDKRPFPKELQNSVLETTLMPHNYSRCYTCQVS NSVSSKNGTVCLSPPCTLARS	IgG1 Fc	HEK293
CD4	MNRGVPFRHLLLVLQLALLPAATQGKKVVLGKKGDTVELTCTASQKKSIQFHWKNSN QIKILGNQGSFLTKGPSKLNDRADSRRSLWDQGNFPLIIKNLKIEDSDTYICEVEDQKE EVQLLVFGLTANSDTHLLQGQSLTLTLESPPGSSPSVQCRSPRGKNIQGGKTLSVSQLE LQDSGTWTCTVLQNQKKVEFKIDIVVLAFQKASSIVYKKEGEQVEFSFPLAFTVEKLTG SGELWWQAERASSSKSWITFDLKNKEVSVKRVTQDPKLQMGKKLPLHLTLPQALPQ YAGSGNLTLALEAKTGKLHQEVNLVVMRATQLQKNLTCEVWGPTSPKLMLSLKLEN KEAKVSKREKAVWVLNPEAGMWQCLLSDSGQVLLESNIKVLPTW	IgG1 Fc	HEK293

Integrin β2	QECTKFKVSSCRECIESGPGCTWCQKLNFTGPGDPDSIRCDTRPQLLMRGCAADDIM<br>DPTSLAETQEDHNGGQKQLSPQKVTLYLRPGQAAAFNVTFRRAKGYPIDLYYLMDLS<br>YSMLDDLRNVKKLGGDLLRALNEITESGRIGFGSFVDKTVLPFVNTHPDKLRNPCPNK<br>EKECQPPFAFRHVLKLTNNSNQFQTEVGKQLISGNLDAPEGGLDAMMQVAACPEEI<br>GWRNVTRLLVFATDDGFHFAGDGKLGAILTPNDGRCHLEDNLYKRSNEFDYPSVGQ<br>LAHKLAENNIQPIFAVTSRMVKTYEKLTEIIPKSAVGELSEDSSNVVQLIKNAYNKLSSR<br>VFLDHNALPDTLKVTYDSFCSNGVTHRNQPRGDCDGVQINVPITFQVKVTATECIQE<br>QSFVIRALGFTDIVTVQVLPQCECRCRDQSRDRSLCHGKGFLECGICRCDTGYIGKNC<br>ECQTQGRSSQELEGSCRKDNNSIICSGLGDCVCGQCLCHTSDVPGKLIYGQYCECDTI<br>NCERYNGQVCGGPGRGLCFCGKCRCHPGFEGSACQCERTTEGCLNPRRVECSGRGR<br>CRCNVCECHSGYQLPLCQECPGCPSPCGKYISCAECLKFEKGPFGKNCSAACPGLQLS<br>NNPVKGRTCKERDSEGCWVAYTLEQQDGMDRYLIYVDESRECVAGPN	His	*E. coli*
Siglec-10	MDGRFWIRVQESVMVPEGLCISVPCSFSYPRQDWTGSTPAYGYWFKAVTETTKGAP<br>VATNHQSREVEMSTRGRFQLTGDPAKGNCSLVIRDAQMQDESQYFFRVERGSYVRY<br>NFMNDGFFLKVTALTQKPDVYIPETLEPGQPVTVICVFNWAFEECPPPSFSWTGAAL<br>SSQGTKPTTSHFSVLSFTPRPQDHNTDLTCHVDFSRKGVSAQRTVRLRVAYAPRDLVI<br>SISRDNTPALEPQPQGNVPYLEAQKGQFLRLLCAADSQPPATLSWVLQNRVLSSSHP<br>WGPRPLGLELPGVKAGDSGRYTCRAENRLGSQQRALDLSVQYPPENLRVMVSQAN<br>RTVLENLGNGTSLPVLEGQSLCLVCVTHSSPPARLSWTQRGQVLSPSQPSDPGVLELP<br>RVQVEHEGEFTCHARHPLGSQHVSLSLSVHYSPKLLGPSCSWEAEGLHCSCSSQASP<br>APSLRWWLGEELLEGNSSQDSFEVTPSSAGPWANSSLSLHGGLSSGLRLRCEAWNV<br>HGAQSGSILQLPDKKGLIST	IgG1 Fc	NS0

Table S3. crRNA sequences

Target	Sequence
CD52 crRNA1	5’ UACCAUAACCAGGAGGCUGA 3’
CD52 crRNA2	5’ CUUAUGUUGCUGGAUGCUGA 3’
Non-targeted control	5’ CGTTAATCGCGTATAATACG 3’

Table S4. CEFT MHC-II peptide sequences

Source	Sequence	Source	Sequence
Cytomegalovirus	DKREMWMACIKELH	Influenza A	PKYVKQNTLKLA
EBV	AGLTLSLLVICSYLFISRG	Influenza A	PKYVKQNTLKLAT
EBV	TSLYNLRRGTALA	Influenza A	DRLRRDQKS
EBV	VPGLYSPCRAFFNKEELL	Influenza A	RGYFKMRTGKSSIMRS
EBV	TGHGARTSTEPTTDY	Influenza B	PYYTGEHAKAIGN
EBV	KELKRQYEKKLRQ	Tetanus	GQIGNDPNRDIL
EBV	TVFYNIPPMPL	Tetanus	QYIKANSKFIGITE
EBV	AEGLRALLARSHVER	Tetanus	QYIKANSKFIGITEL
EBV	PGPLRESIVCYFMVFLQTHI	Tetanus	FNNFTVSFWLRVPKVSASHLE
Influenza A	FVFTLTVPSER	Tetanus	KFIIKRYTPNNEIDSF
Influenza A	SGPLKAEIAQRLEDV	Tetanus	VSIDKFRIFCKALNPK
Influenza A	YDVPDYASLRSLVASS		

Table S5. Markers for the flow cytometry detection of T cell activation and proliferation				
**Laser-Bandpass filter**	**Antigen**	**Fluorochrome**	**Clone**	**RRID**
Violet 405-525/50	CD4 [1]	BV510	SK3	AB_2870492
Blue 488-575/26	CD40L [1]	PE	24-31	AB_2916811
Blue 488-610/20	CD25 [2]	PE-CF594	M-A251	AB_2737636
Blue 488-695/40	HLA-DR [2]	PerCP-eFluor710	L243	AB_10671412
Red 640-670/14	Cell Proliferation Dye (CPD) [1]	eFluor 670	N/A	N/A
Red 640-780/60	Fixable Viability Dye (FVD) [1]	eFluor780	N/A	N/A

1 Becton Dickinson Ltd, Oxford, UK
2 Thermo Fisher Scientific, MA, USA

Table S6. Differentially expressed genes in Regulatory T cells vs. Effector T cells					
**Up-regulation**			**Down-regulation**		
**Gene name**	**Log2 fold change**	**P value**	**Gene name**	**Log2 fold change**	**P value**
FOXP3	127.83	0.00022351	WNT10B	15.37	0.006645918
CTLA4	6.06	0.0049469	CD40LG	6.96	0.001872152
IL1R1	5.43	0.00674576	IL7R	5.21	0.003438049
TIGIT	4.87	0.0098396	TGFB3	4.66	1.712E-05
TNFRSF1B	3.13	0.00367167	CCR7	4.50	0.003821721
CD52	2.92	0.0063044	H2AFY2	3.86	0.002134258
IL2RB	2.63	0.00348238	C5	3.81	0.002749052
IL10RA	2.04	0.00364665	TRADD	2.79	0.000847959
EBI3	1.99	0.00914062	BTLA	2.79	0.006957064
KBTBD8	1.95	0.00280263	TRAT1	2.78	0.00902724
CTSC	1.91	0.00083369	IL6ST	2.54	0.00895103
CEACAM3	1.82	0.00886177	ATM	2.48	0.007328448
AP1S1	1.78	0.00200149	AKT3	2.39	0.004460494
HMGB2	1.7	0.00626315	LTA	2.3	0.005564524
TXN	1.7	0.00723031	IMPDH2	2.15	0.00028604
CASP1	1.69	0.00596212	IKZF1	1.84	0.0006128
TBK1	1.47	0.0006454	RPL4	1.80	0.004097246
RB1	1.4	0.004064134	MTR	1.69	0.004965637
JAK2	1.39	0.003486543	NFATC1	1.62	0.002826721
CSK	1.39	0.006970292	SMN1	1.56	0.000613751
CAPN1	1.31	0.003428262	LPIN1	1.52	0.006860283
			PIK3CD	1.46	0.005215453
			BRAF	1.45	0.002727049
			UBA52	1.45	0.007686436
			EIF4A2	1.45	0.007777643
			IRF3	1.28	0.005928434
			BIRC2	1.16	0.009756252
